# Alterations in estrogen signalling pathways upon acquisition of anthracycline resistance in breast tumor cells

**DOI:** 10.1371/journal.pone.0172244

**Published:** 2017-02-14

**Authors:** Simon Chewchuk, Baoqing Guo, Amadeo Mark Parissenti

**Affiliations:** 1 Ph.D. Program in Biomolecular Science, Laurentian University, Sudbury, ON, Canada; 2 Health Sciences North Research Institute, Sudbury, ON, Canada; 3 Division of Medical Sciences, Northern Ontario School of Medicine, Sudbury, ON, Canada; 4 Division of Oncology, Faculty of Medicine, University of Ottawa, Ottawa, ON, Canada; University of South Alabama Mitchell Cancer Institute, UNITED STATES

## Abstract

Intrinsic or acquired drug resistance is a major impediment to the successful treatment of women with breast cancer using chemotherapy. We have observed that MCF-7 breast tumor cells selected for resistance to doxorubicin or epirubicin (MCF-7_DOX2_ and MCF-7_EPI_ cells, respectively) exhibited increased expression of several members of the aldo-keto reductase (AKR) gene family (in particular *AKR1C3* and *AKR1B10*) relative to control MCF-7_CC_ cells selected by propagation in the absence of drug. Normal cellular roles for the AKRs include the promotion of estrogen (E2) synthesis from estrone (E1) and the hydroxylation and detoxification of exogenous xenobiotics such as anthracycline chemotherapy drugs. While hydroxylation of anthracyclines strongly attenuates their cytotoxicity, it is unclear whether the enhanced AKR expression in the above anthracycline-resistant cells promotes E2 synthesis and/or alterations in E2 signalling pathways and whether such changes contribute to enhanced survival and anthracycline resistance. To determine the role of AKRs and E2 pathways in doxorubicin resistance, we examined changes in the expression of E2-related genes and proteins upon acquisition of doxorubicin resistance. We also assessed the effects of AKR overexpression or downregulation or the effects of activators or inhibitors of E2-dependent pathways on previously acquired resistance to doxorubicin. In this study we observed that the enhanced AKR expression upon acquisition of anthracycline resistance was, in fact, associated with enhanced E2 production. However, the expression of estrogen receptor α (ERα) was reduced by 2- to 5-fold at the gene transcript level and 2- to 20-fold at the protein level upon acquisition of anthracycline resistance. This was accompanied by an even stronger reduction in ERα phosphorylation and activity, including highly suppressed expression of two proteins under E2-dependent control (Bcl-2 and cyclin D1). The diminished Bcl-2 and cyclin D1 expression would be expected to reduce the growth rate of the cells, a hypothesis which was confirmed in subsequent cell proliferation experiments. *AKR1C3* or *AKR1B10* overexpression alone had no effect on doxorubicin sensitivity in MCF-7_CC_ cells, while siRNA-mediated knockdown of *AKR1C3* and/or *AKR1B10* expression had no significant effect on sensitivity to doxorubicin in MCF-7_DOX2_ or MCF-7_EPI_ cells. This suggested that enhanced or reduced AKR expression/activity is insufficient to confer anthracycline resistance or sensitivity to breast tumor cells, respectively. Rather, it would appear that AKR overexpression acts in concert with other proteins to confer anthracycline resistance, including reduced E2-dependent expression of both an important apoptosis inhibitor (Bcl-2) and a key protein associated with activation of cell cycle-dependent kinases (cyclin D1).

## Introduction

Anthracyclines are a class of drugs that are commonly used in adjuvant or neoadjuvant chemotherapy for breast cancer, often in conjunction with other anti-cancer agents [1]. Of this class of chemotherapy agents, doxorubicin or epirubicin are most widely used. Anthracyclines are believed to be cytotoxic to tumor cells through three mechanisms: intercalation between strands of DNA/RNA molecules resulting in interference with normal DNA/RNA synthesis in rapidly dividing cells [2, 3], inhibition of topoisomerase II activity [4], and the creation of iron-mediated oxygen free radicals [5, 6].

Despite their clear utility in the clinical management of breast cancer, many factors negatively affect their efficacy when administered to cancer patients. One such factor is the ability of tumors to resist the cytotoxic action of anthracyclines [7]. This can occur via two distinct mechanisms. First, some tumors exhibit innate resistance to chemotherapy drugs, such that they do not respond to first-line chemotherapy (often referred to as “primary chemotherapy”) [8]. In other instances, patient tumors acquire resistance to anthracyclines and other chemotherapy agents over time. In this latter case, the tumors initially respond partially or almost fully to the administered drugs. However, drug-resistant cells within the tumor cell population survive treatment and continue to replicate, resulting in recurrent disease and disease progression. In some instances, tumors acquire resistance to a wide variety of chemotherapeutic agents, a phenomenon known as multi-drug resistance [7]. Chemo-resistant tumors are usually treated with alternative chemotherapy drugs [9, 10] or alternate downstream treatments such as surgery or radiation therapy [10, 11].

One tool used to study the phenomenon of drug resistance is to look at genotypic and phenotypic changes that take place as tumor cells acquire resistance to chemotherapy drugs in the laboratory. We recently established a panel of MCF-7 breast cancer cell lines, which were selected for survival in increasing concentrations of various chemotherapy agents including the anthracyclines [12]. Microarray studies comparing parental and anthracycline-resistant cells revealed many changes in gene expression accompanying the acquisition of anthracycline resistance, including increased transcripts for several members of the aldo-keto reductase (AKR) family [13] and decreased transcription of genes for estrogen receptor alpha (ERα) and Bcl-2 [13]. The higher levels of expression of AKRs in the above anthracycline-resistant MCF-7 cells relative to drug-sensitive control cells has also been correlated with reduced cellular doxorubicin content, strongly reduced doxorubicin localization to the nucleus, and substantial sequestration of doxorubicin into perinuclear lysosomes [14].

The AKRs are a superfamily of proteins that hydroxylate various endogenous cellular substrates and chemotherapy drugs (reviewed in [15] and [16]). Individual members are identified using a nomenclature method beginning with AKR, followed by a number designating the family, then a letter to denote the sub-family, and finally a number designating the individual member within the sub-family [e.g. *AKR1C3* (for the human gene) or Akr1c3 (for the protein)] [17]. The AKR1 family is the largest of the 15 AKR families and is one of three mammalian AKR families [17]. AKRs are differentially expressed in various tissues throughout the body. *AKR1C1* and *AKR1C4* transcripts have been shown to be primarily expressed in the liver, intestine, mammary glands, prostate, and lungs [17–19]. Akr1c3 is the dominant AKR found in mammary glands. It is also responsible for the hydroxylation of steroid molecules into their active forms; specifically, it converts androstenedione into testosterone and estrone (E1) into estradiol (E2) [20].

E2 is a potent signaling molecule which is active in host tissues and tumors that are positive for estrogen receptors [21–23]. E2 belongs to the E2 family of signaling steroids, which includes the precursor molecules E1 and estriole (E3). Like all steroid molecules, E2 is synthesized from cholesterol [24]. The primary source for E2 in physiological systems differs between males and females. In pre-menopausal women, the majority of E2 is synthesized in the ovaries [25, 26] and serves as the primary source of circulating E2. However, in post-menopausal women and in men, synthesized E2 acts in a paracrine fashion in tissues such as the breast [25]. E2 synthesis is of particular interest in breast cancer studies, due to the effects of E2 on breast epithelial cell proliferation [20, 25, 27] and survival [28] via phosphorylation and activation of ERα[29, 30]. Epidermal growth factor (EGF), a known promoter of cell proliferation is also known to activate ERα phosphorylation [31].

ER signaling has a wide range of effects within cells, including the regulation of transcription [32, 33] and the activation of anti-apoptotic and pro-growth pathways [34, 35]. The genes and pathways affected by E2 vary depending upon which of two E2 receptors is activated (ERα or ERβ) [36]. Both receptors share a common structural homology but differ in size (66 kDa and 56 kDa for ERα and ERβ, respectively) [28, 36].

While ERα expression promotes breast tumor growth, it is generally associated with a favorable outcome for breast cancer patients, as many therapies exist to inhibit ERα function or pathways upstream of this receptor [37–39]. Recently, ERβ has emerged as a possible new prognostic biomarker in breast cancer [40, 41]. Most hormone-dependent cancers rely on locally synthesized hormones for signaling as opposed to systemic hormones [24]. In many cases, the tumors themselves act as the source for these growth-promoting hormones. This is of particular advantage for cancer cells, since it is more energetically favorable to use a local autocrine system to promote growth, as opposed to the higher hormone levels required for endocrine or paracrine systems [24].

In this study, we examined in ER+ MCF-7 breast tumor cells the effects of selection for anthracycline resistance and/or the altered expression of one or more AKRs on E2 metabolism, E2-dependent signaling pathways, expression of E2-dependent genes, cellular growth kinetics, and cellular survival in the presence of anthracyclines. We postulated that, in addition to effects on doxorubicin catabolism and cellular levels of active doxorubicin, the increased expression of AKRs in anthracycline-resistant or AKR-transfected cells will result in higher levels of E2 production and increased cellular proliferation. In addition, we expected to observe increased expression of E2-dependent genes that promote increased tumor cell survival in the presence of anthracyclines.

## Methods

### Culture of wildtype and drug-resistant breast tumour cell lines

The MCF-7 breast cancer cell line was purchased from the American Type Culture Collection (ATCC) and maintained in high glucose D-MEM medium supplemented with 10% FBS, 100 μg/ml streptomycin, and 100 units/ml penicillin (Hyclone) at 37° C in 5% CO_2_. For subculturing, cells in T75 Sarstedt flasks were washed once with sterile PBS followed by the addition of 3 mls of a sterile 0.25% Trypsin, 10 mM EDTA solution (Invitrogen). MCF-7 cells were selected for resistance to increasing doses of either doxorubicin or epirubicin as described previously [12]. Cells at the various selection doses (1 through 12) were stored for subsequent studies. Cells were named based on the selection process, the selection agent and the selection dose. For example, MCF-7_DOX2-9_ cells represent the second time that MCF-7 cells were selected for resistance to doxorubicin up to selection dose 9. Similarly, MCF-7_EPI-12_ cells represent the first time that MCF-7 cells were selected for resistance to epirubicin up to selection dose 12. The various selection doses employed are described in our previously published study [12]. Cells were also selected in the absence of drug to control for genotypic and phenotypic changes due to continued propagation in culture (at identical passage numbers to drug-selected cells). The nomenclature used for these cell lines reflect the absence of drug (co-cultured control or “CC” cells) and the number of passages. For example, MCF-7_CC-12_ cells are cells selected in the absence of drug for an equivalent number of passages as anthracycline-selected cells (selected to dose 12). Drug-resistant cells were maintained in media supplemented with the corresponding selection dose of doxorubicin or epirubicin. They were, however, placed in drug-free medium media for 3–4 days prior to use in experiments.

### Dextran charcoal filtering of cell culture media

To remove endogenous steroid compounds and growth factors, D-MEM media supplemented with 10% FBS and antibiotics were treated with 0.5% (w/v) dextran-coated charcoal (Sigma Chemical, St. Louis, MO), and heated to 50°C with constant shaking for 30 min. The charcoal-treated media was then sterile-filtered using a Sarstedt 0.22 μm, 500 ml vacuum filter. Dextran charcoal-treated D-MEM media (DC-DMEM) were supplemented with 10^−7^ M E2 for ER signalling experiments, or 10^−7^ M estrone (E1) for E1 metabolic experiments (Sigma Chemical).

### Assessment of cellular E2 levels

Estradiol (E2) levels in wildtype and drug-resistant cells were determined using a standard enzyme-linked immunosorbent assay (ELISA). Briefly, 5x10^5^ cells/well were plated in 6 well plates (Sarstedt), in D-MEM media supplemented with 10% FBS and antibiotics and allowed to adhere overnight. D-MEM media was replaced with dextran/charcoal-stripped D-MEM medium (DC-DMEM) 18 hours after plating. Cells were treated with one of: a vehicle control solution (5% DMSO), 10^−7^ M E1, 10^−7^ M E1 with 5x10^-9^ M Letrozole (aromatase inhibitor), or 10^−7^ M E1 (E1) with 0.2 mM β-cholanic acid (in 5% DMSO) (Akr1c3 inhibitor). Aliquots of media were collected 24h post treatment and stored at -20°C. E2 levels in the media were then assayed using E2 competitive ELISA kits (US Biological Life Sciences) as per the manufacturer’s protocol.

### Assessment of cellular sensitivity to anthracyclines using clonogenic assays

The sensitivity of the above-described cell lines to various doses of anthracyclines was determined using a clonogenic assay, which monitors the ability of drug-treated cells to form colonies in drug-free semi-solid methylcellulose medium, as previously described in [13]. The mean number of viable colonies was determined in 12 randomly selected fields ± the standard error of the mean. Each clonogenic experiment was repeated twice.

### Creation of a mammalian *AKR1C3* expression plasmid and transfection in MCF-7 cells

A plasmid (pENTR211) containing the open reading frame (ORF) for the *AKR1C3* gene was purchased from Invitrogen (Burlington, ON) and transfected into *E*. *coli* DH5α cells using standard methods. Successful transformants were cultured on LB-Agar plates containing 50 μg/ml of kanamycin as the selective agent. Plasmid DNA from a positive bacterial clone (propagated in kanamycin-containing LB medium) was isolated using QIAprep miniprep plasmid extraction kits (Qiagen). The ORF for *AKR1C3* was amplified by PCR using the purified plasmid as template and the following AKR1C3-specific primers [Forward: 5’- GCT AAG ATC TTC ATG GAT TCC AAA CAC CAG TGT G -3’, Reverse: 5’- TCG ACT CGA GGT ACA AGA AAG CTG GGT TCT AAT ATT CAT C -3’ (IDT)]. After cleavage with appropriate restriction endonucleases, the *AKR1C3* cDNA within the amplified ORF product was ligated into identical cleavage sites within the pCMV-FLAG plasmid (Stratagene), such that the FLAG Tag was incorporated in frame with the C-terminal domain of the protein-coding sequence. The ligation reaction was transformed into competent DH5-α *E*. *coli* bacteria and cultured on kanamycin-containing plates to select for clones harboring the pAKR1C3-FLAG plasmid. The recombinant plasmid was purified from bacterial transformants using Qiagen QIAprep maxiprep kits (Qiagen). Plasmid concentrations and purity were determined by monitoring absorbance at 260 nm and 280 nm. The AKR1C3-Tag insert within pAKR1C3-FLAG was sequenced (MOBIX) to confirm that no mutations were introduced into the AKR sequence during DNA cloning.

Clones of cells stably expressing the AKR1C3 cDNA were generated by transfecting MCF-7_CC-12_ cells with pAKR1C3-FLAG DNA using Lipofectamine 2000 and Optimem^TM^ media (Invitrogen). Cells were incubated overnight, and then subjected to selective screening in D-MEM medium containing 2.0 mg/ml G418 (Sigma) until G418-resistant clones were obtained. Cells transiently overexpressing *AKR1C3* were also generated by transfection with 24 μg of plasmid DNA complexed to 60 μl of Lipofectamine 2000. The cells were transfected for 18 h and then re-plated for subsequent experiments. The amount of overexpression of Akr1c3 protein was assessed in immunoblotting experiments employing an anti-human Akr1c3 antibody (Sigma) using the protocol described below.

### siRNA-mediated knockdown of *AKR1C3* and *AKR1B10* expression

Silencer select siRNA oligoribonucleotides were obtained from Life Technologies (Burlington, ON) to induce the specific degradation of *AKR1C3* and *AKR1B10* transcripts. Two different siRNAs were used to reduce expression of these genes [16448 (KD1) and 225013 (KD2) for AKR1C3, and 32583 (KD3) and 32584 (KD4) for AKR1B10]. In addition, a scrambled control siRNA (SCRAM, also from Life Technologies) was also used as a negative control to identify any possible off-target effects, including those induced by the transfection process itself. Cells were transfected with 5 ng of siRNA using Lipofectamine 2000, as per the manufacturer’s directions. Knockdowns were confirmed by quantitative PCR and western blot analyses. The effect of the siRNAs on cellular sensitivity to anthracyclines was also assessed using clonogenic assays.

### Immunoblotting experiments with epitope- and phospho-specific antibodies

Cells were lysed by incubation in standard RIPA buffer supplemented with 1x Complete protease inhibitor cocktail (Roche), 10 μM NaF, 2 mM Na_3_VO_4_ and 1 μM PMSF (Sigma). The amount of protein in each whole cell extract was quantified using the BCA assay, as per the manufacturer’s protocol (Pierce). Extracts were aliquoted into 1.5 ml tubes containing standard Laemmeli’s sample buffer (1X final concentration) and boiled for 5 min, after which the proteins in the extracts were resolved by standard SDS acrylamide gel electrophoresis procedures. The resolved proteins in the extracts were transferred by electrophoresis to nitrocellulose membranes and the membranes probed with specific primary antibodies and appropriate HRP-conjugated secondary antibodies using standard immunblotting procedures. In some instances, blots were stripped for 1 h at 50°C in a buffer containing 6.24 mM Tris pH 6.7, 2% (w/v) SDS, and 100 mM β-mercaptoethanol before re-probing with another antibody.

### Assessment of estrogen receptor alpha and estrogen receptor beta transcript expression

The level of human ERα gene (*ESR1*) transcripts in the above cell lines was assessed by quantitative PCR (Q-PCR). Qiagen RNeasy kits (Qiagen) were used to extract total RNA from the above cell lines. Prior to Q-PCR, all RNA samples were assessed for RNA integrity by capillary electrophoresis on an Agilent 2100 Bioanalyzer using RNA 6000 Nano Assay Lab Chips as per the manufacturer’s directions (Agilent Technologies). RNA to be used in Q-PCR experiments had to exhibit an RNA integrity number (RIN) of ≥ 8 and a 28S/18S ratio of ≥ 1.8. All RNA samples were subjected to DNase treatment prior to reverse transcription as previously described [42]. The expression of *ESR1* transcripts was then assessed by Q-PCR as described [42]. The *ESR1* primers used were (Forward: 5’-CCACCAACCAGTGCACCATT-3’, Reverse: 5’-GGTCTTTTCGATTCCCACCTTTC-3’). All RNA samples were assessed for *ESR1* transcript expression in triplicate with the transcript for the S28 ribosomal protein as the reference gene.

### Immunohistochemical assessment of estrogen receptor alpha protein expression

The expression of human ERα protein expression in the above cell lines was assessed by immunohistochemistry with a human ERα antibody. Cells were harvested and the cell pellets, collected after centrifugation, were delivered on ice to the Pathology Department of Health Sciences North, Sudbury, ON. Cell pellets were fixed in a 5% (v/v) formalin solution for 24 h. The samples were then embedded in Histogel^TM^ prior to embedding in paraffin blocks. Histological slices were taken from the paraffin blocks and prepared for Hematotoxin and Eosin (H&E) staining for visualization of tumor cells and immunohistochemical staining using an HRP-conjugated human ERα antibody.

### Quantitative determination of cellular levels of activated ERα

Levels of active (nuclear) ERα in cells were quantified using human ERα TransAM™ kits purchased from Active Motif (Carlsbad, CA), using the manufacturer’s protocol.

### Artemisinin-induced reduction in cellular ERα protein expression

In some experiments, cellular ERα protein expression was strongly reduced by their incubation with Artemisinin (Sigma). This enabled us to assess changes in cellular phenotypes associated with strongly reduced ERα levels. Cells were plated at 30% confluence in 10 cm plates containing D-MEM supplemented with 10% FBS and antibiotics. After cells had adhered for 24 h, the media was removed and replaced with D-MEM supplemented with 10% FBS and either 300 μM Artemisinin or 0.1% v/v DMSO as the control. Cells were allowed to grow at 37°C at 5% CO_2_ for 72 h. Following the treatment period, proteins were extracted from the cells as previously described.

### Data analysis

All graphs were prepared using GraphPad Prism V5.0 software. All data points depicted represent the mean ± standard error of mean (SEM). Statistical analyses were performed using GraphPad Prism V 5.0 software. Analysis of Variance (ANOVA) tests were performed, assuming a normal distribution for all data sets, followed by a Bonferoni post hoc test for significance. Comparisons were made between drug-selected and co-cultured control cell lines at various selection doses, as well as between treated and untreated cell lines or between cells expressing and not expressing specific transcripts or siRNAs. Due to the multi-parametric nature of the data analyses, an ANOVA was chosen as the most suitable method of data analysis. Differences between samples or cell lines were deemed to be significant if they had a p value of < 0.05 for the above statistical tests.

## Results

### Expression of AKR1C isoforms

Previously conducted DNA microarray studies comparing gene expression between MCF-7_CC-12_ and MCF-7_DOX2-12_ cells identified a number of differences in gene expression that accompany the acquisition of doxorubicin resistance [13]. Among these were members of the AKR 1C gene family, although the probes used in the microarray experiments could not effectively distinguish between the various transcripts of AKR 1C isoforms. Subsequent Q-PCR experiments using isoform-specific primers confirmed that both *AKR1C2* and *AKR1C3* transcripts are elevated in MCF-7_EPI-12_ and MCF-7_DOX2-12_ cell lines (compared to MCF-7_CC-12_ cells). *AKR1C3* transcript levels were 11.9 ± 3.9-fold higher in MCF-7_DOX2-12_ cells line (p<0.0001) and 4.5 ± 0.9-fold higher in MCF-7_EPI-12_ cells compared to the MCF-7_CC-12_ cells. Similarly, *AKR1C2* transcript levels were 5.8 ± 1.1-fold and 4.6 ± 1.2-fold higher in MCF-7_EPI-12_ and MCF-7_DOX2-12_ cells compared to MCF-7_CC-12_ cells, respectively [13].

**Fig 1:**
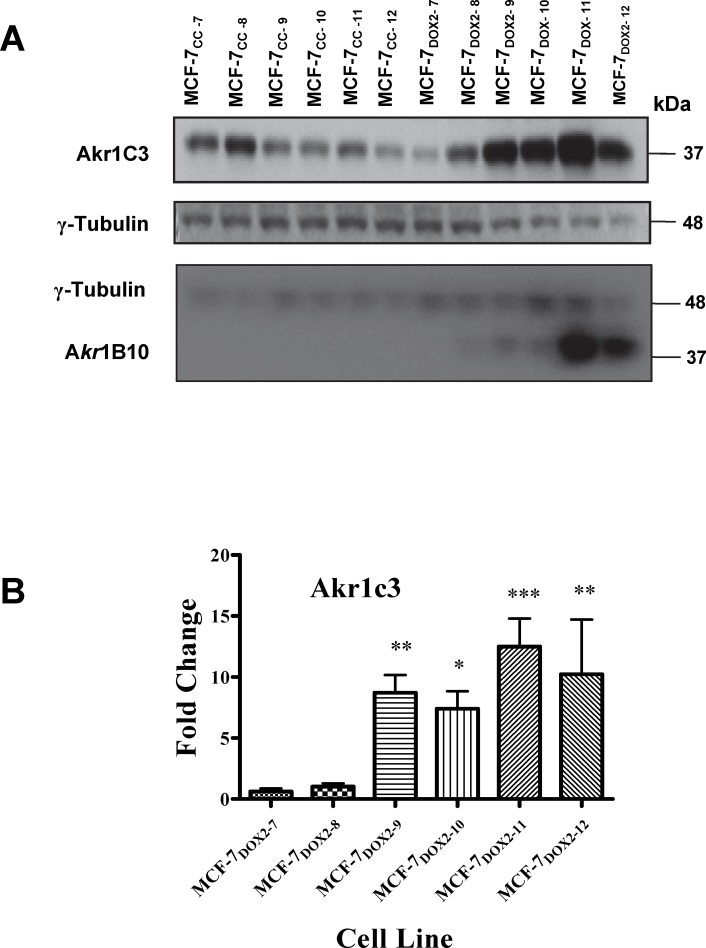
Assessment of Akr1c3 and Akr1b10 and γ-tubulin expression in various stable cell lines using immunblotting approaches. **(A)** Representative immunoblots for assessing Akr1c3, Akr1b10 and γ-tubulin protein expression in extracts of unselected MCF-7_CC_ cells or doxorubicin-selected MCF-7_DOX2_ cells (selection doses 7 through 12). Blots represent one 4 independent experiments. **(B)** Fold change in Akr1C3 levels relative to corresponding co-cultured control cell lines for doxorubicin-selected cell lines across selection doses 7 through 12 (based by densitometry). Fold changes are expressed as the average ± S.E.M. for 4 independent experiments. The significance of differences in Akr1c3 expression between the designated doxorubicin-elected cell line and its corresponding co-cultured control cell line was assessed using an ANOVA test, followed by a Bonferoni correction. * p< 0.05, ** p< 0.01, *** p< 0.001, **** p< 0.0001.

Given that *AKR1C3* was the most strongly upregulated aldo-keto reductase gene in the anthracycline-resistant cell lines, we recently assessed protein extracts of the above cell lines across the various selection doses for their level of Akr1c3 protein expression, as measured using standard immunoblotting procedures (Fig 1A). As shown in Fig 1B, no statistically significant differences in Akr1c3 expression were observed between MCF-7_DOX2-7_ or MCF-7_DOX2-8_ cells and their co-cultured control cell lines (MCF-7_CC-7_ and MCF-7_CC-8_ cells). However, beginning at selection dose 9 (MCF-7_DOX2-9_), we observed an 8.7±1.5 fold (p≤ 0.01) induction in Akr1c3 protein expression relative to its co-cultured control cell line. MCF-7_DOX2-10_ cells showed a 7.4±1.4 fold (p≤ 0.05) induction, while cells selected to doses 11 and 12 showed 12.5 ± 2.3-fold and 10.2 ± 4.5-fold inductions, (p≤ 0.001 and p≤ 0.01, respectively; Fig 1B). Interestingly, the expression of another AKR isoform not involved in estrogen biosynthesis (Akr1b10) also increased in a dose-dependent manner as the doxorubicin selection dose was increased beyond selection dose 8 (Fig 1A). In contrast, continued propagation of MCF-7 cells in the absence of doxorubicin (MCF-7_CC_ cells) resulted in reduced Akr1c3 protein expression, suggesting that cell propagation under optimal cellular conditions in the absence of a cell stressor (such as doxorubicin) reduces the expression of aldo-keto reductases.

### Transient and stable overexpression of Akr1c3 protein in MCF-7_CC-12_ cells

**Fig 2:**
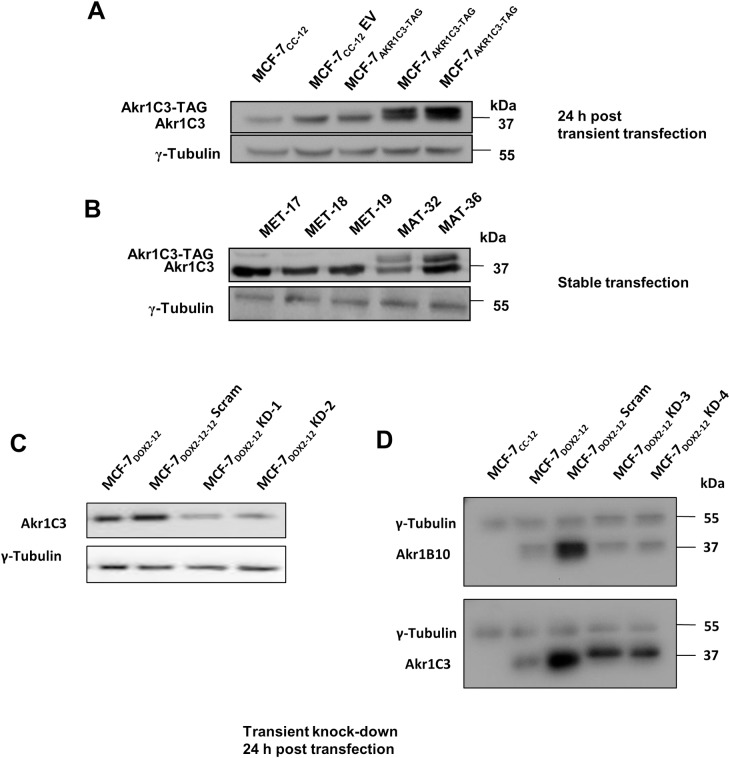
Assessment of Akr1c3, Akr1b10 and γ-tubulin expression in various stably and transiently transfected cell lines using immunblotting approaches. A) Optimization of transfection using lipofectamine 2000. Lane 1 represents untreated MCF-7_CC-12_ cells, lane 2 is for identical cells transfected with an empty vector (pCMV-FLAG) in the presence of lipofectamine (1:2 ratio), while lanes 3, 4 and 5 are cells transfected with FLAG-tagged AKR1c3 expression vector (pCMV-AKR1C3-FLAG) at vector to lipofectamine ratios of 1:1, 1:1.5 and 1:2, respectively. B) A representative western blot assessing Akr1C3 protein expression in MCF-7_CC-12_ cells stably transfected with an empty vector (lanes 1, 2 and 3) or pAKR1C3-FLAG (lanes 4 and 5). C) A representative western blot assessing Akr1c3 protein expression in cells transfected with a random RNA sequence (scrambled) or with an *AKR1C3*-specific siRNAs (KD-1 and KD-2). D) A representative western blot assessing Akr1b10 and Akr1c3 protein expression in cells transfected with a scrambled siRNA or *AKR1B10*-specific siRNAs (KD-3 and KD-4).

We then assessed whether stable or transient expression of recombinant *AKR1C3* transcripts in MCF-7_CC-12_ cells could, by itself, induce doxorubicin resistance. Fig 2 depicts the level of Akr1c3 protein expression in untreated MCF-7_CC-12_ cells, MCF-7_CC-12_ cells exposed to transfection conditions with DNA empty vector control, and MCF-7_CC-12_ cells transiently (panel A) and stably (panel B) transfected with a human *AKR1C3* expression vector (pCMV-AKR1C3-FLAG). Cellular levels of Akr1c3 protein were determined in immunoblotting experiments using an Akr1c3-specific antibody, with a γ-tubulin antibody being used as a loading control. After densitometric quantification of the Akr1c3 bands, the levels of total Akr1c3 expression relative to MCF-7_CC-12_ cells was then determined for the transiently and stably transfected cells. We found that while transient transfections did not result in permanent elevations in Akr1c3 expression, the fold increase in total Akr1c3 immunoreactivity was much greater (up to 5-fold) for transiently transfected MCF-7_CC-12_ cells than stable transformants (up to 1.7-fold). It should be noted that during optimization experiments, Akr1c3 levels were found to increase when cells were treated with an empty vector (pCMV-FLAG; see Fig 2A), suggesting that the stresses associated with the transfection process are sufficient to induce Akr1c3 expression. This was confirmed in subsequent experiments where cells transfected under identical conditions in the absence of expression plasmids also exhibited increased Akr1c3 expression (data not shown).

### Effect of Akr1c3 overexpression on cellular doxorubicin sensitivity

**Fig 3:**
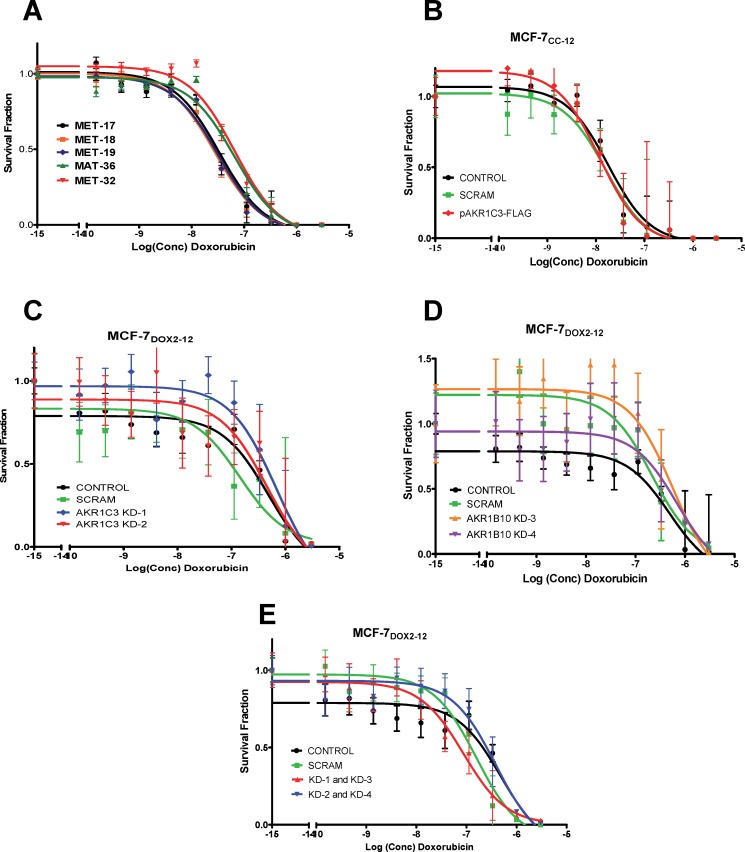
Differences in sensitivity to doxorubicin, as measured using clonogenic assays for MCF-7_CC-12_ cells stably or transiently transfected with various constructs. Survival curves are depicted for: **(A)** stable clones of MCF-7_CC-12_ cells transfected with pAKR1C3-FLAG, **(B)** MCF-7_CC-12_ cells, with or without transient transfection with the pAKR1C3-FLAG plasmid, **(C)** MCF-7_DOX2-12_ cells, with or without transient transfection with scrambled or *AKR1C3*-specific siRNAs, **(D)** MCF-7_DOX2-12_ cells, with or without transient transfection with scrambled or *AKR1B10*-specific siRNAs, **(E)** MCF-7_DOX2-12_ cells, with or without transient transfection with scrambled or *both AKR1C3*- and *AKR1B10*-specific siRNAs.

We then assessed whether the transient or stable expression of recombinant Akr1c3 alters the sensitivity of MCF-7_CC-12_ cells to doxorubicin using a clonogenic assay. The two clones having the highest stable expression of recombinant Akr1c3 (MAT36 and MAT32, Fig 2A) had a higher IC_50_ for doxorubicin (110 ± 93 nM) than stable clones with little to no Akr1C3 expression (46 ± 29 nM) (see Fig 3A). This translates into a 2.4-fold decrease in doxorubicin sensitivity, consistent with a possible role for Akr1c3 in doxorubicin resistance; however, this difference was not deemed to be significant using an ANOVA. Perhaps the modest fold increase (1.5-fold) in total Akr1C3 expression was insufficient to confer a statistically significant reduction in doxorubicin sensitivity. We thus examined whether the ~5-fold change in total Akr1C3 expression found in MCF-7_CC-12_ cells transiently transfected with pCMV-AKR1C3-FLAG might be sufficient to induce statistically significant changes in doxorubicin sensitivity. As shown in Fig 3B, no significant change in doxorubicin sensitivity was observed. The IC_50_ for doxorubicin in untransfected MCF-77_CC-12_ cells was 16.2 nM, while mock-transfected and pCMV-AKR1C3-FLAG-transfected cells had IC_50_s for doxorubicin of 14.4 nM and 12.7 nM, respectively. Taken together, the data suggests that simply increasing the expression of one AKR isoform (Akr1c3) was insufficient to confer doxorubicin resistance in MCF-7_CC-12_ cells.

In order to further assess the relationship between the expression of AKRs and doxorubicin resistance, we then assessed the effect of *AKR1C3*- and *AKR1B10*-specific siRNAs on doxorubicin sensitivity in MCF-7_DOX2-12_ cells. siRNAs against *AKR1C3* were designated KD1 and KD2, while siRNAs against *AKR1B10* were designated KD3 and KD4. Interestingly, transfection of MCF-7_DOX2-12_ cells with a scrambled control siRNA strongly increased both Akr1c3 and Akr1b10 expression (Fig 2D), similar to our observations of increased Akr1c3 expression in MCF-7_CC-12_ cells upon transfection with an empty vector (pCMV-FLAG; Fig 2A). Nevertheless, transient transfection of MCF-7_DOX2-12_ cells with the *AKR1C3*- or *AKR1B10*-specific siRNAs resulted in an average reduction in Akr1c3 or Akr1b10 expression of 50 to 80% relative to the scrambled control siRNA (Fig 2C and 2D, respectively). The KD-1 and KD-3 siRNAs gave the highest knockdown of Akr1c3 and Akr1b10 protein expression, respectively (at near 80%; Fig 2C and 2D). Despite the high data variability, the IC_50_s for doxorubicin in MCF-7_DOX2-12_ cells transfected with the *AKR1C3* siRNAs (680 nM and 460 nM), were very similar to that of untransfected MCF-7_DOX2-12_ cells (490 nM; see Fig 3C). Similarly, knockdown of Akr1b10 expression in MCF-7_DOX2-12_ cells resulted in IC_50_s for doxorubicin of 560 nM and 690 nM for the two siRNAs (Fig 3D), very similar to that of MCF-7_DOX2-12_ cells. Knockdown of one of the AKR isoforms did not induce an upregulation of the expression of the other AKR isoform (Fig 2D). To address possible effects of compensatory gains in the expression of one Akr isoform due to the loss of another isoform, we performed a dual-knockdown experiment in which we treated cells with *both AKR1C3*- and *AKR1B10*-specific siRNAs (KD1 and KD3 or KD2 and KD4). Transfection of MCF-7_DOX2-12_ cells with both siRNAs did not result in any significant change in doxorubicin sensitivity (Fig 3E).

### Factors affecting E1 to E2 conversion in various breast tumor cell lines

**Fig 4:**
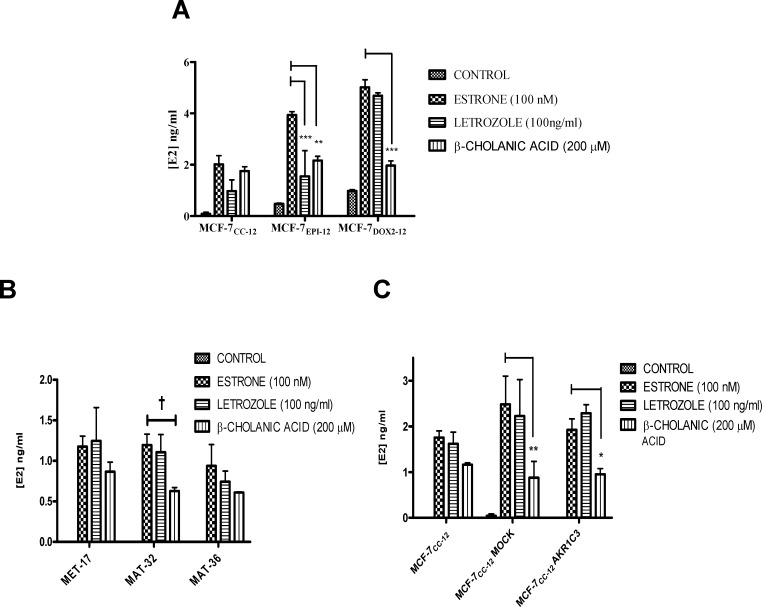
Degree of Production of Estradiol (E2) from Estrone (E1) in various cell lines under varying conditions. **(A)** MCF-7_DOX2-12_, MCF-7_EPI-12_, and MCF-7_CC-12_ cells. **(B)** Clones of MCF-7_CC-12_ cells stably transfected with pAKR1C3-FLAG. **(C)** MCF-7_CC-12_ cells, with or without transient transfection with pAKR1C3-FLAG. Some cells were pre-treated with an AKR inhibitor (β-cholanic acid) or an aromatase inhibitor (Letrozole) for 1 h prior to the addition of 100 nM estrone. Estradiol levels were quantified using E2 ELISA kits. Bars represent the mean from 3 independent trials with technical duplicates ± S.E.M. Statistical analysis was done using an ANOVA test, followed by a Bonferoni correction. * p< 0.05, ** p< 0.01, *** p< 0.001.

Since Akr1C3 is known to play a role in E1 to E2 conversion, the above-documented increases in Akr1C3 expression in MCF-7_DOX2_ cells and MCF-7_EPI_ cells would be expected to result in higher cellular E2 levels. Given that E2 promotes cell growth and survival in breast tumor cells [43, 44], the elevated E2 levels could, in turn, contribute to the observed anthracycline resistance phenotype. To assess the former hypothesis, MCF-7_CC-12_, MCF-7_DOX2-12_, and MCF-7_EPI-12_ cells (selected to dose level 12) were treated with exogenous E1 (100 nM) and the amount of E2 released in the medium determined in the presence or absence of various pathway inhibitors. These included letrozole (which inhibits the aromatization of androgens androstenedione and testosterone to estrone and estradiol, respectively) and β-cholanic acid, a known AKR inhibitor. E2 secreted into the media of cells was monitored over a 24 h period. By inhibiting the aromatase pathway we were able to monitor the effects of β-cholanic acid on the direct conversion of E1 to E2. As shown in Fig 4A, even in the absence of exogenously added E1, both MCF-7_DOX2-12_ and MCF-7_EPI-12_ cells secreted elevated levels of E2 in the medium relative to MCF-7_CC-12_ cells. This is consistent with the induction of Akr1c3 and the enzyme’s ability to promote the conversion of endogenous E1 to E2. Upon addition of exogenous E1, media levels of E2 dramatically increased in all examined cell lines (MCF-7_CC-12_, MCF-7_EPI-12_ and MCF-7_DOX2-12_ cells). Pre-treatment of cells with the aromatase inhibitor 1etrozole (100 ng/ml) for 1 h prior to the addition of 0.1 μM exogenous E1 reduced E2 secretion into media, but this reduction was only significant for MCF-7_EPI-12_ cells (p≤ 0.001). Pre-treatment of cells with the AKR inhibitor β-cholanic acid (200 μM) for 1 h prior to the addition of E1 had little effect on media E2 levels for MCF-7_CC-12_ cells (1.8±0.2 ng/ml), consistent with the considerably lower levels of Akr1C3 expression in MCF-7_CC-12_ cells compared to MCF-7_EPI-12_ and MCF-7_DOX2-12_ cells. In contrast, the latter two cell lines exhibited significant reductions in media E2 levels in the presence of β-cholanic acid (p≤ 0.01 and p≤ 0.001, respectively).

Stable Akr1c3-FLAG-expressing clones (MET-17, MAT-32, and MAT-36) were analyzed for E2 levels in the absence or presence of E1 and/or pharmacological inhibitors of aromatase or Akr1c3. While all stable transfectants exhibited dramatically enhanced E2 production in the presence of exogenous E1, there were no differences in E2 production among the E1-treated stable transfectants. The addition of letrozole had no effect on E1 to E2 conversion, while β–cholanic acid treatment consistently reduced E1 to E2 conversion in stably transfected cells, but these reductions were not statistically significant using an ANOVA test (Fig 4B). Treatment with E1 also induced strong E1 to E2 conversion in transiently transfected cells, and even in untransfected and mock-transfected cells (Fig 4C). Similar to the stably transfected cells, addition of letrozole had no effect on the conversion of E1 to E2. In contrast, β–cholanic acid treatment significantly reduced E1 to E2 conversion in mock (p≤ 0.01) and pAKR1C3-FLAG-transfected cells (p≤ 0.05). Untransfected wildtype cells also exhibited a reduction in E2 production, but this was not significant using an ANOVA test (Fig 4C). As only Akr1C3 is known to convert E1 to E2 conversion, Akr1B10 overexpressing constructs were not assessed for their effects on E2 metabolism.

### Changes in ERα transcript levels upon selection for anthracycline resistance

**Fig 5:**
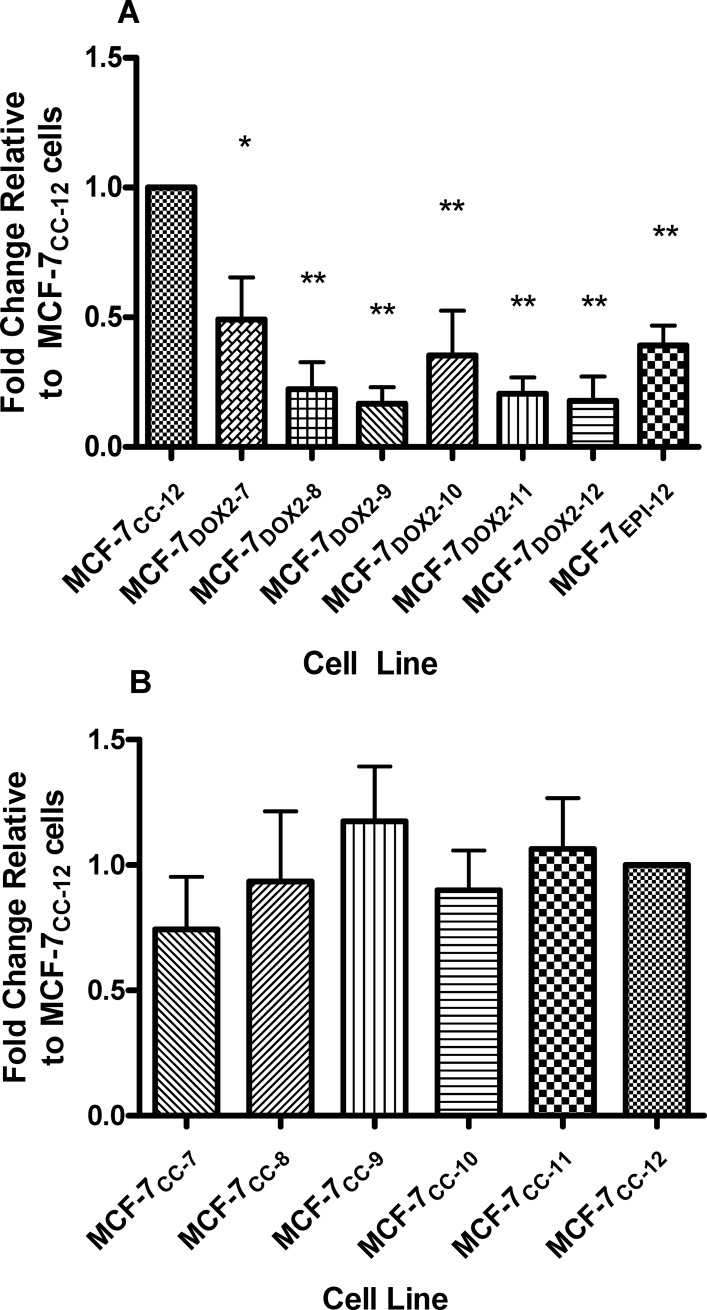
Differences in *ESR1* transcript levels in various cell lines, as determined by quantitative PCR (Q-PCR). (**A)** *ESR1* transcript levels in MCF-7 cells selected for survival in increasing concentrations (doses) of doxorubicin or epirubicin (relative to MCF-7_CC-12_ cells). Data for doxorubicin selection doses 7 through 12 and for epirubicin at selection dose 12 are depicted. **(B)** A similar approach was used to quantify *ESR1* transcript levels in MCF-7 cells selected in the absence of drug to similar passage numbers as MCF-7_DOX2_ cells (selected to dose levels 7 through 12. All *ESR1* expression values were normalized to the expression of transcripts for ribosomal protein S28. Values depicted represent the mean ± S.E.M. for three independent trials. The significance of differences in *ESR1* transcript levels between the designated cell line and MCF-7_CC-12_ cells was assessed using an ANOVA test, followed by Bonferoni correction. n = 3, * p< 0.05, ** p< 0.001

The elevated E2 production in anthracycline-resistant cell lines would be expected to result in their enhanced proliferation [20, 25, 27] through E2’s ability to bind to and activate ERα[45, 46]. This is providing ERα expression and activity is unaltered upon selection for anthracycline resistance. We thus examined the level of expression of ERα transcripts throughout selection of MCF-7 cells for survival in increasing concentrations (doses) of doxorubicin or epirubicin. As shown in Fig 5A, during selection for doxorubicin resistance, dose 7 cells exhibited a 2.0 ± 0.2-fold (p≤ 0.05) reduction in ERα transcript (*ESR1*) levels compared to MCF-7_CC-12_ cells. As selection progressed through doses 8 through 12, *ESR1* transcript levels further decreased by up to 6-fold (p≤ 0.01 for doses 8–12). MCF-7 cells selected for resistance to epirubicin (MCF-7_EPI-12_ cells) also showed a 2.6 ± 0.1-fold (p≤ 0.01) reduction in *ESR1* levels relative to MCF-7_CC-12_ cells (Fig 5A). All Q-PCR Ct values were normalized to the expression of the S28 reference gene. All fold changes in transcript levels were expressed relative to levels in MCF-7_CC-12_ cells. No significant differences in ERα transcript levels were observed upon selection in the absence of doxorubicin or epirubicin (MCF-7_CC_ selection doses 7 through 12; Fig 5B).

### Estrogen receptor expression and activity levels in anthracycline-resistant cells

**Fig 6:**
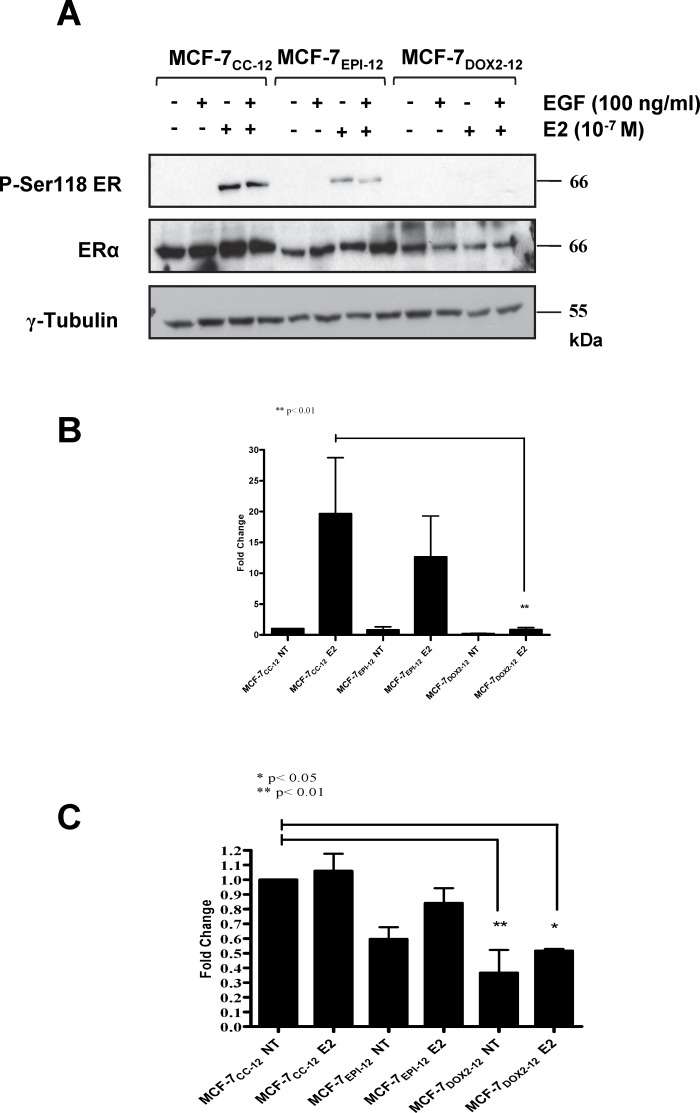
Differences in the expression of estrogen receptor alpha (ERα), phosphorylated estrogen receptor alpha at serine 118 (P-Ser118 ER), and γ tubulin in various cell lines, as measured in immunoblotting experiments with epitope- or phospho-specific antibodies. **(A)** Immunoblots were conducted using extracts of cells with or without incubation in the presence of 100 nM E2, 100 mg/ml epidermal growth factor (EGF), or a combination of E2 and EGF. Primary antibodies purchased from Cell Signaling and used at 1:1000 dilution in 0.5% BSA overnight at 4°C. **(B)** Fold change in Ser-118 phosphorylated estrogen receptor relative to untreated MCF-7_CC-12_ cells, normalized to γ-tubulin expression (left) and to basal ERα expression (right). Data is expressed as the mean fold change observed in 3 independent experiments (± S.E.M., with the value of untreated MCF-7_CC-12_ cells set to 1.0. **(C)** Fold change in ERα levels, in a manner identical to that of P-Ser118 ER. The significance of differences between the test sample and that of untreated MCF-7_CC-12_ cells was assessed using an ANOVA test, followed by Bonferoni correction. * = p< 0.05, ** = p< 0.001

We then conducted immunoblotting experiments to assess changes in ERα protein levels upon selection for anthracycline resistance (Fig 6A and 6C). Consistent with *ESR1* transcript levels, we did observe reductions in ERα protein expression in both MCF-7_EPI-12_ and MCF-7_DOX2-12_ cells relative to MCF-7_CC-12_ cells, although only the decrease in MCF-7_DOX2-12_ cells was found to be statistically significant using an ANOVA test (p<0.001). Interestingly, as shown in S1 Fig, no changes in the expression of estrogen receptor beta (ER-β) were observed during selection for doxorubicin resistance (selection doses 7 through 12).

The observation that E1 to E2 conversion was increased in MCF-7_EPI-12_ and MCF-7_DOX2-12_ cells, prompted an analysis of whether this was associated with increased levels of phosphorylated (active) ERα, as determined using an antibody that binds to ERα when phosphorylated at Ser118. Cells were serum-starved overnight and then treated in hormone-depleted media for 30 min with either 100 nM E2, 100 ng/ml EGF, or a combination of the two agents. Previously published studies have shown that E2 and EGF are strong activators of ERα phosphorylation [29, 31, 44]. As shown in Fig 6A, E2 (but not EGF) was able to strongly induce ERα phosphorylation in MCF-7_CC-12_ cells. Interestingly, ERα phosphorylation was undetectable and considerably lower in MCF-7_DOX2-12_ and MCF-7_EPI-12_ cells, respectively, relative to that seen in MCF-7_CC-12_ cells (Fig 6A). Only levels of phosphorylated ERα in MCF-7_DOX2-12_ cells were found to be significantly reduced relative to MCF-7_CC-12_ cells in the presence of exogenous E2 (p≤ 0.01)_._ This finding held true even when levels of phosphorylated estrogen receptor were standardized to total estrogen receptor (Fig 6B). In three independent experiments, treatment of MCF-7_CC-12_ cells with E2, EGF or a combination of the two had little to no effect on ERα receptor levels. However, both MCF-7_EPI-12_ and MCF-7_DOX2-12_ cells showed overall reductions in ERα receptor levels.

**Fig 7:**
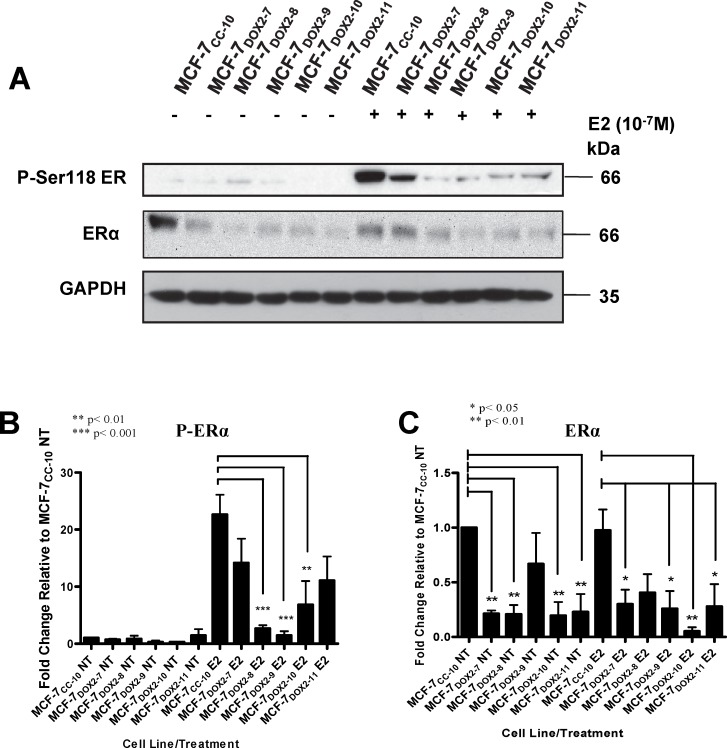
Differences in the expression of estrogen receptor alpha (ERa), phosphorylated estrogen receptor alpha at serine 118 (P-Ser118 ER), and GAPDH during selection for survival in the absence or presence of increasing concentrations (doses) of doxorubicin (selection doses 7 through 11). Immunoblots were conducted using extracts of cells without or with a treatment with 100 nM E2. Primary antibodies purchased from Cell Signaling and used at 1:1000 dilution in 0.5% BSA overnight at 4°C. **(A)** Representative western blots for Era and GAPDH. **(B)** Data for P-Ser118 ER is expressed relative to untreated (NT) MCF-7_CC-12_ cells. **(C)** Data for ERa is expressed relative to untreated (NT) MCF-7_CC-12_ cells. All expression values were first normalized to GAPDH expression. Bars represent the mean ± S.E.M. for three independent experiments. The significance of differences between the test sample and that of treated or untreated MCF-7_CC-12_ cells was assessed using an ANOVA test, followed by Bonferoni correction. * p< 0.05,** p< 0.01, *** p< 0.001

In subsequent experiments (Fig 7), we examined changes in ERα receptor levels during selection for doxorubicin resistance (selection doses 7 to 11). Cells exhibited reductions in both phosphorylated ERα and total ERα levels during selection for doxorubicin resistance, particularly the former. Reductions in total ERα expression relative to untreated MCF-7_CC-10_ were first observed as early as selection dose 7 relative to untreated co-culture control cells. This was mirrored by a similar reduction in ERα levels in cells treated with E2. The total ERα levels continued to decline at higher selection doses (Fig 7). Reductions in P-Ser118-ERα levels across the selection doses mirrored that observed above for ERα levels with significant reductions observed at doses 8 and 9 (p≤ 0.001) and dose 10 (p≤ 0.01), when compared to MCF-7_CC-10_ cells treated with E2.

### Changes in ERα transcriptional activity in anthracycline resistance

**Fig 8:**
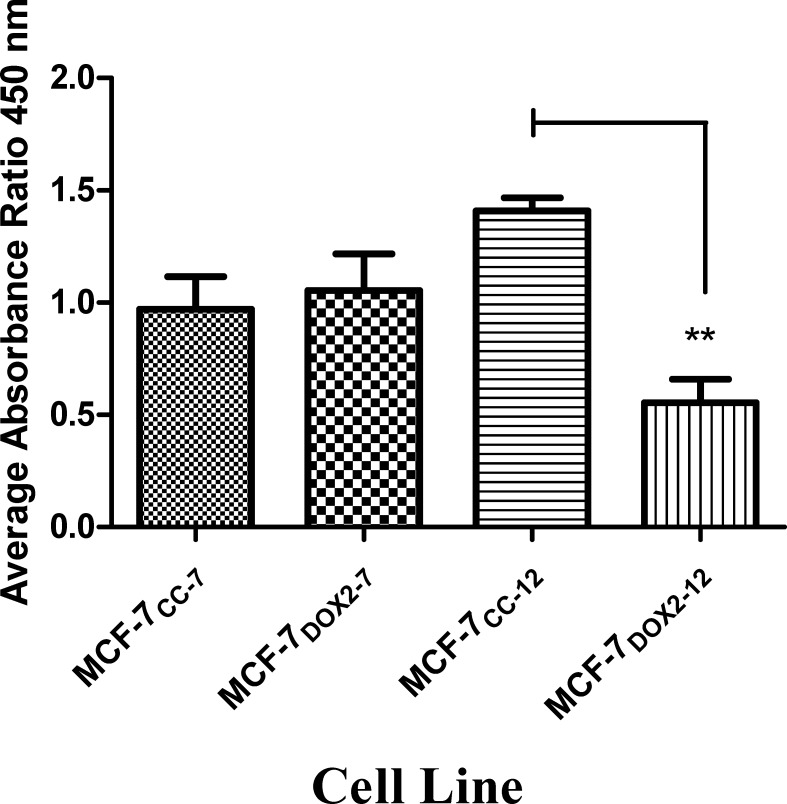
Relative levels of active ERα in nuclear extracts. Active Era levels were measured in MCF-7_CC_ cells and MCF-7_DOX2_ cells at selection doses 7 and 12, as determined by ERa TRANS-AM kits. Absorbances were corrected for background and expressed as a ratio (450 nM to 655 nm). Extracts were performed under basal conditions with cells grown in D-MEM to 90% confluence. Values depicted are the mean ± S.E.M. of three independent experiments, each made up of a duplicate technical replicate. The significance of differences in active ERα levels between samples was assessed using an ANOVA test, followed by Bonferoni testing. n = 3, ** p< 0.01

The observed reductions in ERα expression and phosphorylation (activation) upon selection for doxorubicin resistance would be expected to result in reduced ERα activity, as assessed by the receptor’s ability to bind the E2 response element (ERE). For this assessment a TransAM^TM^ kit from Active Motif, Inc. (Carlsbad, CA) was used, where the amount of ERα binding to the ERE sequence in the presence of E2 was expressed as the corrected absorbance at 450 nm (after substraction of background) divided by the corrected absorbance at 655 nm (after subtraction of background). As shown in Fig 8, MCF-7_CC_ and MCF-7_DOX2_ cells at selection dose 7 had very similar absorbance ratios. However, at selection dose 12 (where significant doxorubicin resistance is obtained), the absorbance ratio for MCF-7_CC_ cells increased, while the absorbance for MCF-7_DOX2-12_ cells decreased. This represented roughly a significant 2.6-fold reduction in the level of active ERα in the nucleus of MCF-7_DOX2-12_ cells (p≤ 0.01).

**Fig 9:**
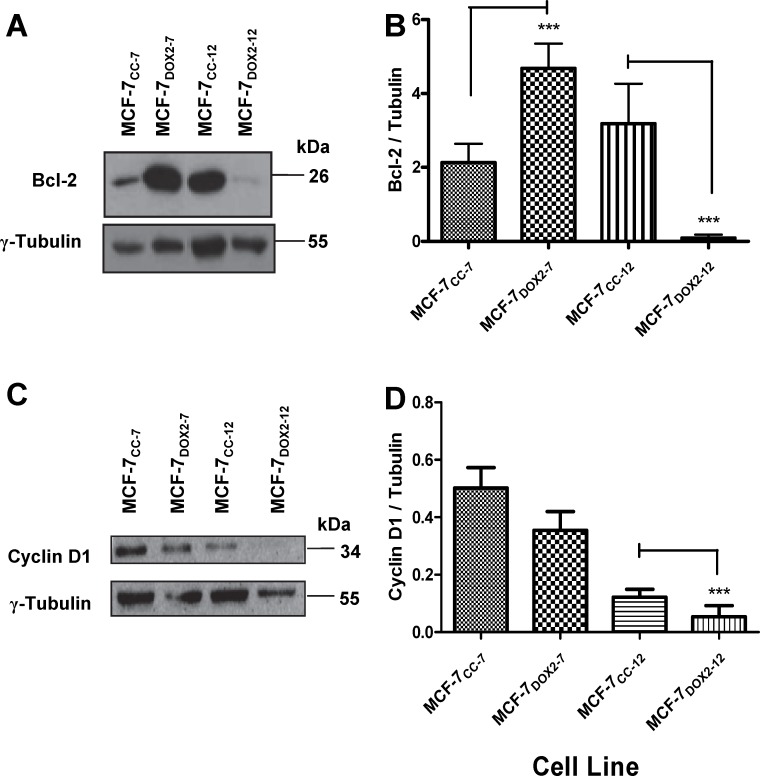
Expression of Bcl-2, cyclin D1, and γ tubulin proteins in various cell lines, as determined in immunoblotting experiments with epitope-specific antibodies. **(A)** A representative western blot for Bcl-2 expression in MCF-7_CC_ and MCF-7_DOX2_ cells at selection doses 7 and 12. Results shown are representative of 3 independent trials. **(B)** Fold change in Bcl-2 levels in MCF-7_DOX2_ cells (relative to the appropriate co-cultured control cell line). Results shown are the average of 3 independent trials. **(C)** A representative western blot for cyclin D1 expression in MCF-7_CC_ and MCF-7_DOX2_ cells at selection doses 7 and 12. Results shown are representative of 3 independent trials. **(D)** Fold change in cyclin D1 levels in MCF-7_DOX2_ cells (relative to the appropriate co-cultured control cell line). Results shown are normalized to the expression of γ tubulin and are the average of 3 independent trials. The significance of differences in expression levels for Bcl-2 and cyclin D1 between samples was assessed using an ANOVA test, followed by Bonferoni testing. n = 3, *** p< 0.001

The reduction in the ability of ERα to bind the ERE sequence in MCF-7_DOX2_ cells would be expected to result in a concomitant reduction in the expression of genes whose expression is positively regulated by ERα namely Bcl2 and cyclin D1 [45, 47, 48]. We thus examined in immunoblotting experiments the expression of Bcl-2 and Cyclin D1in MCF-7_CC_ and MCF-7_DOX2_ cells at selection doses 7 and 12 using antibodies that specifically recognize these proteins (Fig 9A and 9C, respectively). Expression of these proteins was quantified by densitometry and expressed relative to that of a reference protein (γ tubulin). As shown in Fig 9A and 9B, Bcl-2 levels were significantly higher (p≤ 0.001) in MCF-7_DOX2_ cells at selection dose 7 compared to MCF-7_CC_ cells at similar passage number (MCF-7_CC_ cells at dose 7). The elevated expression of this apoptosis inhibitor may have helped facilitate resistance to doxorubicin at the early selection doses. However, Bcl-2 levels were dramatically reduced in MCF-7_DOX2_ cells as selection progressed to dose level 12 compared to MCF-7_CC-12_ cells (p≤ 0.001). This would be consistent with the reduced and dramatically reduced levels of ERα and phosphorylated ERα in MCF-7_DOX2-12_ cells relative to MCF-7_CC-12_ cells, respectively (Fig 6). Cyclin D1 levels were slightly reduced upon selection for doxorubicin resistance to dose level 7 (MCF-7_DOX2-7_ cells) compared to MCF-7_CC-7_ cells. At selection dose 12, however, the difference in cyclin D1 expression between MCF-7_DOX2-12_ and MCF-7_CC-12_ cells became significantly different (p≤ 0.001) and could be due to reduced levels of ERα and phosphorylated ERα in MCF-7_DOX2-12_ cells. Interesting, the expression of a number of ER-β-dependent genes (CCNA1, HSD11B2, and TMOD1), as measured by quantitative PCR, was not significantly changes during selection for doxorubicin resistance (S1 Fig).

### Knock-down of ERα expression with artemisinin

**Fig 10:**
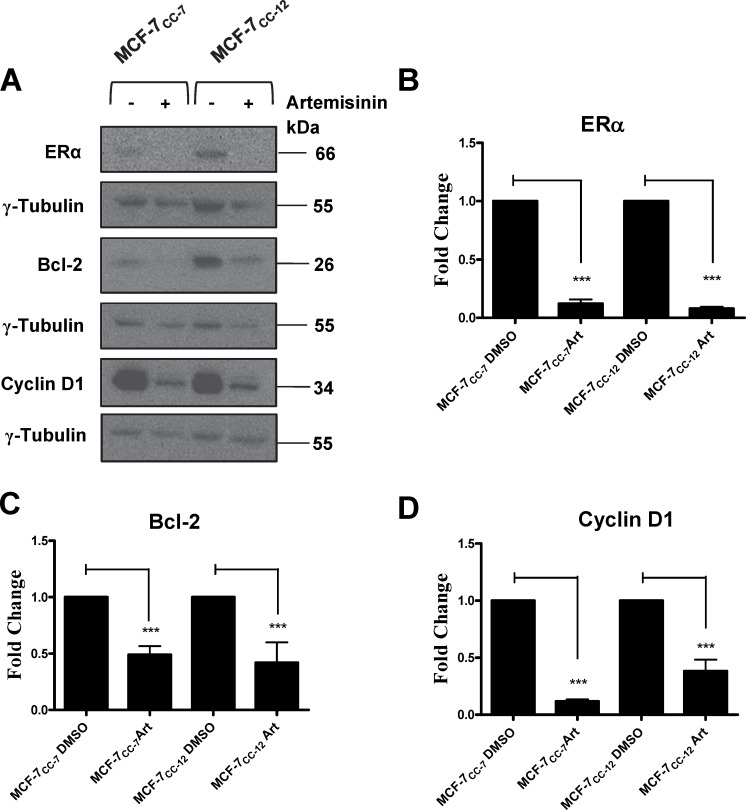
Artemisinin-mediated knockdown of ERα expression and its consequent effects on Bcl-2 and cyclin D1 expression in MCF-7_CC-7_ and MCF-7_CC-12_ cells. **(A)** MCF-7_CC-7_ and MCF-7_CC-12_ cells were treated with either DMSO or 300 μM artemisinin. Whole cell extracts of these cells were then monitored for Bcl-2, cyclin D1, and γ tubulin protein expression using immunoblotting approaches with epitope-specific antibodies. γ tubulin was used as loading control. Blots are representative of 3 independent trials. **(B)** Fold changes in ERα levels induced by artemisinin in MCF-7_CC_ cells at selection doses 7 and 12. **(C)** Fold changes in Bcl-2 levels induced by artemisinin in MCF-7_CC_ cells at selection doses 7 and 12. **(D)** Fold changes in cyclin D1 levels induced by artemisinin in MCF-7_CC_ cells at selection doses 7 and 12. All values depicted are the mean ± S.E.M. for 3 independent experiments. The significance of artemisinin-induced changes in the expression of the above proteins was assessed using an ANOVA test, followed by a Bonferoni correction. n = 3, *** p< 0.001

In order to assess the relationship between estrogen receptor signalling and the expression of estrogen-dependent genes involved in cellular growth and survival, we initially chose to knock down ERα expression using an siRNA approach. As shown in Supplemental Fig 2, the selected siRNAs only reduced ERα transcript expression by approximately half. This level of ERα transcript suppression had no significant effect on doxorubicin sensitivity (S2 Fig) or the expression of ERα-dependent genes. As an alternate approach, MCF-7_CC_ cells were treated for 72 hours with 300 μM artemisinin. Artemisinin is a known potent blocker of ERα gene transcription and it would be expected that the expression of ERα-regulated genes such as Bcl2 and cyclin D1 would be strong affected by artemisinin (despite the lack of doxorubicin resistance in MCF-7_CC_ cells). Since artemisinin has been shown to be toxic to cells, we used the maximum concentration of artemisinin that had no effect on cell growth. Fig 10A shows representative immunoblots for ERα, Bcl-2, and Cyclin D1 in the absence or presence of artemisinin, with densitometry values for the expression of ERα, Bcl-2, and cyclin D1 in various cell lines normalized to the expression of γ-tubulin depicted in Fig 10B, 10C and 10D, respectively. As expected, artemisinin treatment resulted in a clear reduction in the expression of ERα and concomitant reductions in the expression of Bcl-2 and cyclin D1 (p<0.001 for all observations).

**Fig 11:**
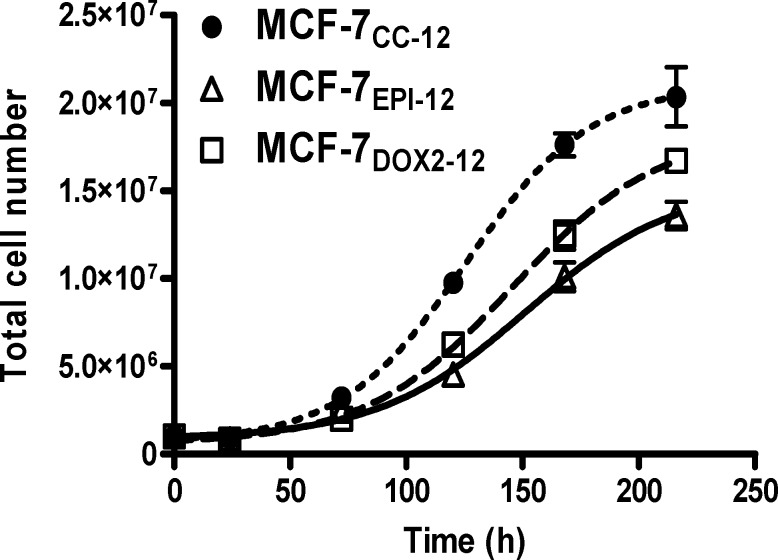
Cell growth curves for MCF-7_CC-12_, MCF-7_DOX2-12_, and MCF-7_EPI-12_ cells. Exponentially growing cells were counted using a hemocytometer and introduced into T75 flasks at a density of 10^6^ cells per 10 ml of D-MEM medium. Specific growth plots were generated using Graphpad Prism 5.0, modelled after the Gompertz growth equation. n = 3

The changes in expression of cyclin D1 would be expected to have an influence on the proliferation rate of cells. To ascertain if doxorubicin-resistant cells had differing rates of cell division compared to wildtype cells, MCF-7_CC-12_, MCF-7_DOX2-12_, and MCF-7_EPI-12_ cells were plated in 10 cm plates at low densities (10^6^ cells per plate) and allowed to grow until saturation. Cells were counted at daily intervals. It was observed that MCF-7_CC2-12_ cells had a maximum growth rate of 180,000 cells h^-1^, while MCF-7_DOX2-12_ and MCF-7_EPI-12_ cells had maximum growth rates of 130,000 cells h^-1^ and 104,000 cells h^-1^, respectively (Fig 11). Comparisons of curves generated by the Gompertz equation showed that the maximum specific growth rates differed significantly between all three cell lines (p<0.001).

## Discussion/Conclusions

Resistance to cytotoxic chemotherapy drugs can be the result of several factors working in collaboration to protect tumor cells from drug-induced death. Previous microarray experiments comparing gene expression between the wildtype and the anthracycline-resistant MCF-7 cell lines used in this study (MCF-7_DOX2_ and MCF-7_EPI_ cells), revealed a number of gene expression changes associated with the acquisition of anthracycline resistance [13]. Many of these changes in gene expression were subsequently validated using Q-PCR. Among the more prominent genes whose expression was altered upon acquisition of anthracycline resistance were members of the ATP-binding cassette (ABC) family of drug transporters as well as drug metabolizing proteins such as the AKRs. Coupled with findings from a previously published study [12] and a subsequent study [14], it became clear that two of the largest changes in gene expression for MCF-7_EPI_ cells were the Abcb1 drug transporter (a known efflux transporter of epirubicin) and the aldo-keto reductase Akr1c2 (or genes highly homologous to it). For MCF-7_DOX2_ cells, three of the largest changes in gene expression were the Abcc1 drug transporter (a known efflux transporter of doxorubicin) and both Akr1c3 and Akr1b10. The above ABC drug transporters would be expected to actively excrete anthracyclines from tumor cells, thereby reducing their accumulation in tumor cells and their cytotoxicity. Supporting this view, the uptake of doxorubicin into MCF-7_DOX2-12_ and MCF-7_EPI-12_ was found to be significantly lower than that of MCF-7_CC-12_ cells [12]. The ability of the aldoketoreductases to hydroxylate and inactivate anthracyclines would also be expected to contribute to anthracycline resistance. However, Akr1c3 has a clear preference for hydroxylation of idarubicin and daunorubicin over doxorubicin [49, 50]. Moreover, while Akr1C3 was able to hydroxylate doxorubicin as a purified protein, these studies were unable to demonstrate the ability of Akr1c3 to hydroxylate doxorubicin within cells.

In the current study, we specifically examined the role that the overexpression of AKR proteins may play in E2 metabolism in MCF-7_DOX2_ and MCF-7_EPI_ cells and whether alterations in E2 metabolism may also contribute to the anthracycline-resistant phenotype in these cells.

### Akr1c3 expression and function in anthracycline resistant MCF-7 Cells

In the present study, we confirmed the overexpression of Akr1c3 protein in MCF-7_DOX2_ cells, with MCF-7_DOX2-11_ cells having the highest expression of Akr1c3 gene and protein expression (Fig 1A). This increase in Akr1c3 expression occurred upon acquisition of anthracycline resistance and the level of expression of Akr1c3 increased with increasing resistance to doxorubicin (Fig 1A). Due to the relationship between increased Akr1c3 expression and elevated E2 synthesis from E1 [20, 51], we theorized that the higher cellular expression of Akr1c3 could potentially have broad impacts on E2-dependent signaling pathways that may also contribute to anthracycline resistance. Specifically, it was expected that the increased Akr1C3 expression would promote E2 synthesis from E1, which in turn would activate ERα-dependent survival pathways through either the non-genomic pathway via AKT or through the genomic pathway by increasing expression of Bcl-2 [30, 52, 53]. Such events would perhaps lead to enhanced survival and growth in the presence of anthracyclines (see further discussion below). While we observed that Akr1B10 levels in MCF-7 cells were dramatically increased upon selection for doxorubicin resistance (Fig 1A), it is important to note that there is no published evidence that Akr1B10’s promotes estrogen biosynthesis from estrone.

We subsequently demonstrated in this study that the increased expression of Akr1C3 in doxorubicin-selected cells (to dose level 12) did have a dramatic effect on the cells’ ability to synthesize and secrete E2. As expected, cells that had higher levels of Akr1C3 expression also exhibited higher levels of basal and E1-induced E2 synthesis and excretion (Fig 4A). This supports the idea that increased levels of Akr1C3 expression could be related to cell survival in chemotherapy resistant cell lines by promoting elevated production of E2 and subsequent activation of ERα-dependent growth and survival pathways, including the activation of AKT and the inhibition of apoptosis [30, 48, 54].

### Overexpression of Akr1c3, by itself, does not confer anthracycline resistance

To assess whether elevated expression of Akr1c3, by itself, could induce resistance to doxorubicin, we stably or transiently transfected MCF-7 cells with a vector for the constitutive expression of Akr1c3. Elevated levels of Akr1c3 expression relative to mock-transfected cells were observed in these transformants (Fig 2). Specifically, it was observed that transient transfection resulted in a greater induction of Akr1c3 protein expression (relative to mock transfected cells) than stably transfected cells (Fig 2C and 2D). Clonogenic assays performed on stably selected clones showed on average of a 2.4 fold increase in resistance to doxorubicin relative to empty vector controls (Fig 2A), but this difference was not found to be statistically significant in an ANOVA test. This increase in drug resistance was unlikely to be biologically relevant, since even a higher level of Akr1c3 induction in transiently transfected cells resulted in no change in doxorubicin sensitivity (Fig 2B).

### *AKR1C3* knockdown in doxorubicin-resistant cells did not restore doxorubicin sensitivity

Since overexpression of Akr1c3 transcripts and protein in MCF-7_CC-12_ cells showed little ability to induce doxorubicin resistance, we attempted to determine if elevated levels of *AKR1C3* and/or *AKR1B10* transcripts were required to maintain resistance in MCF-7_DOX2-12_ cells. Knockdown experiments were conducted using two different siRNAs targeting different regions of the *AKR1C3* or *AKR1B10* mRNAs. In addition, a scrambled control siRNA was used, to determine if any off-target effects and/or the process of transfection affected cellular sensitivity to doxorubicin. While individual transfections with the siRNAs resulted in as much as an 80% knockdown of Akr1c3 protein expression in the cells (Fig 2), no significant change in doxorubicin sensitivity was observed in the presence of either siRNA (Fig 3C and 3D). To rule out compensatory effects with the individual knockdowns, we proceeded to knock down both Akr1c3 and Akr1B10 expression simultaneously by co-transfecting MCF-7_DOX2-12_ cells with both siRNAs. Knockdown with both siRNAs achieved the same level of knockdown of their respective RNAs as the individual transfections (up to 80%). However, no significant change in doxorubicin sensitivity was observed (Fig 3E). To assess whether the effects of the *AKR1C3* and *AKR1B10* siRNAs are specific for their respective transcripts (with no effect on other AKR transcripts), we examined siRNA-transfected cells for expression of Akr1c3 and Akr1b10 proteins. We observed only minor changes in Akr1c3 levels in cells transfected with the *AKR1B10* siRNA (despite dramatically reduced Akr1b10 levels). Reduced expression of the target transcripts was verified in all siRNA experiments using western blotting experiments with Akr1c3 and Akr1b10 antibodies. Images in Fig 2 are representative of these controls. The observation of a lack of effect of Akr1c3 knockdown on doxorubicin resistance suggests that the AKRs, while upregulated in doxorubicin resistance, did not actually contribute to the drug resistant phenotype. Supporting this view, Hoffman et al. [49] observed that while Akr1c3 can convert doxorubicin to the less toxic doxorubicinol in a cell-free system, it has a relatively low affinity for doxorubicin and could not hydroxylate doxorubicin in *AKR1C3*-transfected cells. It should be noted that this is in contrast to studies involving the overexpression of an *AKR1C3* cDNA into cancer cells. A recent study by Zhong et al showed that overexpression of recombinant Akr1c3 in MCF-7 cells increased their resistance to doxorubicin [55]. While our observations differ from these findings, we suggest that the differences observed are due to the level of overexpression. We were only able to increase the Akr1C3 level in MCF-7 cells by a maximum of 7-fold using lipofectimine 2000. In contrast, Zhong et al. were able to overexpress Akr1C3 by nearly 200 fold over non-transfected cells using a viral expression vector [55]. It is possible that there is a minimum threshold of over-expression required in order for Akr1C3 to promote estrogen production and/or have a significant impact on cell survival in the presence of doxorubicin. Moreover, this very high level of Akr1c3 overexpression may permit doxorubicin hydroxylation in cells, despite its poor affinity for doxorubicin as a substrate in cells [49, 50]. The higher level of Akr1c3 expression in MCF-7_DOX2_ cells appears to be clearly sufficient to promote E2 biosynthesis and the activation of ER-dependent survival pathways. This may be particularly relevant at lower selection doses, where induction of the ABC transport proteins is minimal or much lower [12]. We therefore postulate that in the MCF-7_DOX2_ cell model we present here, the AKRs contribute to the resistance phenotype. However, selection for survival in the presence of doxorubicin typically involves selection for multiple resistance mechanisms. Our previous studies have shown that MCF-7_DOX2-12_ cells strongly overexpress the Abcc1 drug transporter [12]. Moreover, we have recently observed that the Abcc1 inhibitor MK-571 can partially restore the sensitivity of MCF-7_DOX2-12_ cells to doxorubicin (Chewchuk et al., manuscript in preparation).

### E2 biosynthesis and cellular growth rates in anthracycline-resistant cells

We next determined whether the overexpression of Akr1c3 in anthracycline-resistant cells was associated with alterations in E2 biosynthesis and cellular growth rate. Stably transfected clones showed no significant difference in E1 to E2 conversion between cells transfected with an “empty vector” and *AKR1C3*-transfected cells (Fig 4B). This suggested that the level of increase in *AKR1C3* expression in the stable transformants was insufficient to impact on E2 biosynthesis. Only a slight reduction in E1 to E2 conversion was observed when cells *stably* transfected with the *AKR1C3* expression vector were treated with β-cholanic acid, but not in the empty vector controls (Fig 4B). This difference was statistically significant for clone MAT-32 using a Student t-test, but not by the more stringent ANOVA. However, β-cholanic acid was without effect when added to cells of the MAT-36 stable cone, which exhibited the highest expression level of Akr1c3 (Figs 2B and 4B). Taken together our observations suggest that Akr1c3 overexpression is typically insufficient in the stable clones to promote additional E2 biosynthesis, which could be inhibited by β-cholanic acid.

When cells *transiently* transfected with the *AKR1C3* vector were assessed, no statistically significant increase in E2 biosynthesis was observed using an ANOVA or Student t-test (Fig 4C), including cells transfected with an empty vector (mock-transfected cells). This was despite the increased expression of endogenous and FLAG-tagged recombinant Akr1c3 observed in these cells (Fig 2A). However, a strong, highly significant reduction in E2 synthesis was observed upon treatment of empty vector- or *AKR1C3*-transfected cells with β-cholanic acid (Fig 4C). This was likely due to the effects of β-cholanic acid on both endogenous and/or recombinant Akr1c3 expression observed during transfection of MCF-7 cells with the empty vector or *AKR1C3* expression plasmid (Fig 2A and 2C).

In contrast to the above findings, E2 biosynthesis was considerably higher in drug-resistant MCF-7_DOX2-12_ and MCF-7_EPI-12_ cells than drug-sensitive MCF-7_CC-12_ cells. (Fig 4A) This biosynthesis in the anthracycline-resistant cell lines was strongly inhibited by both the aromatase inhibitor letrazole and the aldo-keto reductase inhibitor β-cholanic acid. The considerably higher levels of E2 production in the resistant cell lines are likely due to their higher level of overexpression of Akr1c3 compared to MCF-7_CC-12_ cells and MCF-7_CC-12_ cells stably or transiently transfected with the *AKR1C3* expression vector. These findings are consistent with those recently published by Byrns et al. [56]. It may be possible to further increase the overexpression of Akr1c3 to more closely match the levels observed in the MCF-7_DOX2_ and MCF-7_EPI_ cells lines (possibly by other transfection methods such as electroporation or a retroviral vector. By increasing the overexpression of Akr1c3 to match more closely those of the chemotherapy drug resistant cell lines it may still be possible to induce doxorubicin resistance. However, more likely, the increase in Akr1c3 expression in transient or stable transfectants was insufficient to induce doxorubicin resistance.

### Effects of selection for anthracycline resistance on cellular ERα expression

E2 is a known promoter of growth in breast tumor cells [20, 25, 27]. Thus, it would be expected that the elevated production of E2 in MCF-7_DOX2-12_ and MCF-7_EPI-12_ cells would result in higher growth rates for these cells relative to the co-cultured control cell lines. However, we observed in this study a reduction in growth rates for MCF-7_DOX2_ and MCF-7_EPI_ cells compared to MCF-7_CC_ cells (Fig 11). It is therefore likely that some defect in the E2 signaling pathway exists in the anthracycline-resistant cells that prevents the elevated E2 levels from promoting growth. We thus looked at the levels of ERα and phosphorylated ERα (at Ser118) in the above cell lines in the absence or presence of estrogen and/or EGF. It has been reported that the phosphorylation of ERα on Ser118 is critical for the function of the non-genomic E2 signaling pathway, which regulates cell proliferation through effects on ERK and/or Bcl-2 survival pathways [34, 43, 54]. We observed that while EGF had no effect on ERα phosphorylation, MCF-7_CC-12_ cells treated with 100 nM E2 exhibited dramatic increases in ERα phosphorylation on Ser118 residues. Phosphorylation was substantially lower in MCF-7_EPI-12_ cells and undetectable in the MCF-7_DOX2-12_ cells (Fig 6A and 6B). This was not expected since MCF-7 cells are normally ER positive and have been shown to require E2 signaling for normal growth [43]. Subsequent immunoblotting experiments using an antibody to ERα revealed that the reduced phosphorylation was due to, at least in part, a downregulation of ERα expression in the anthracycline-resistant cell lines, in particular for MCF-7_DOX2-12_ cells (Fig 6A and 6C). The reduced ERα expression appeared to be due to reduced levels of *ESR1* transcripts (Fig 5). The complete lack of detection of ERα phosphorylation for MCF-7_DOX2-12_ cells, suggests a complete downregulation of E2’s ability to phosphorylate any available ERα as some ERα expression is still evident in these cells; see Fig 6.

We then hypothesized that upon selection for anthracycline resistance, the increased production of E2 via Akr1C3 overexpression activated a compensatory negative feedback loop to reduce ERα synthesis. However, subsequent experiments revealed that the reduction in ERα transcript levels occurred prior to AKR1c3 upregulation (compare Figs 1A and 5A). This would argue against a negative feedback mechanism as the increase in E2 concentration would not have occurred to induce the progressive loss of ERα. The mechanisms for the reduced expression of ERα in MCF-7_DOX2_ and MCF-7_EPI_ cells relative to MCF-7_CC_ cells have yet to be elucidated, but may include: increased methylation of CpG islands or decreased histone acetylation within the *ESR1* promoter, resulting in reduced gene transcription (epigenetic changes), reduction in the activities of transcription factors associated with ERα expression such as the AP-1 transcription factors Fos and Jun [28], or reductions in ERα transcript stability.

It is interesting to note that changes in tumor ERα expression have been observed after neoadjuvant chemotherapy for patients with breast cancer, although the percentage of patients exhibiting a loss of tumor ERα expression post-chemotherapy was found to be only 6% [57]. This is in contrast to 19% of patients losing tumor progesterone receptor (PR) expression after chemotherapy [57]. While there are some clear limitations associated with this study of 368 non-randomized patients, it is possible that such losses in tumor ERα expression may be associated with resistance to anthracycline-based chemotherapy. The loss of tumor ERα expression status after neoadjuvant chemotherapy (if validated in future studies) would have very strong implications in terms of patient care. Patients with tumors lacking ERα expression post-chemotherapy would likely be unresponsive to endocrine therapies targeting estrogen signaling pathways, such as tamoxifen and exemestane [58, 59].

### Effects of ERα downregulation on the growth of anthracycline-resistant cells

The growth rate of the anthracycline-resistant cells at selection dose 12 was assessed, since ERα has been documented to regulate cell growth in breast tumor cells [34]. It was observed that both MCF-7_DOX2-12_ and MCF-7_EPI-12_ cells exhibited significantly reduced growth rates in comparison to MCF-7_CC_ cells (Fig 11). This could be due to downregulation of ERα activity in these cells, although other pathways may also have been impacted on cellular growth rate. Despite this discrepancy, both cell lines exhibited lower growth rates which correlated with the loss of ERα expression and function. This observation led to the hypothesis that in acquiring resistance to chemotherapy, MCF-7 cells were selected for variants in the population with a slower growth rate. This could impart a survival advantage by allowing the cells more time to repair any cellular damage, before activation of cell death pathways. In addition, many chemotherapy agents such as doxorubicin selectively target rapidly dividing cells. In this light, it is reasonable to expect a growth promoting signaling protein, such as ERα, to be downregulated upon selection of breast tumour cells for anthracycline resistance.

### Changes in the expression of ERα-specific genes upon acquisition of anthracycline resistance

The reduction in ERα expression would be expected to strongly impact the expression of estrogen-dependent genes, in particular if this resulted in a change in the ability of the receptor to bind to the estrogen response element (ERE) regulating gene expression. To assess this, we first examined whether nuclear extracts from MCF-7_DOX_ cells exhibited a lower ability to bind the ERE than nuclear extracts from MCF-7_CC_ cells using the ERα TRANS Am kit. Nuclear extracts taken from MCF-7_CC-7_ and MCF-7_DOX2-7_ cells showed no statistically significant differences in the amount of active nuclear ERα (ERE binding) between the two cell lines. This was despite the reduced total levels of ERα in MCF-7_DOX2-7_ cells at this selection dose observed in western blotting experiments. This could indicate that while the total amount of ERα in the cells is reduced, the proportion of ERα that is active changes to maintain the basal requirements of the cells. However, MCF-7_DOX2-12_ cells showed a significant reduction in active ERα in nuclear extracts relative to MCF-7_CC-12_ cells. This suggests that ER activity is also suppressed at high selection doses (consistent with the reduced phosphorylation of ERα), which involves more than the downregulation of ERα protein expression. Consistent with the abolished activity of ERα in MCF-7 cells, we observed reduced expression of two genes, whose expression is strong upregulated by ERα, namely Bcl-2 and cyclin D1 [30, 60] (Fig 10). Both genes possess EREs in their promoter regions [36], and have been shown to be highly responsive to E2 treatments [45, 48, 60]. Bcl-2 levels were significantly elevated in MCF-7_DOX2-7_ cells relative to co-cultured controls cell lines As Bcl-2 is an anti-apoptotic/pro-survival protein, it stands to reason that the elevated levels of Bcl-2 would be observed early in the selection for doxorubicin resistance. However, it should be noted that in previous studies conducted in our laboratory [13], doxorubicin resistance was only achieved when the doxorubicin selection dose reached 29.1 nM (dose 9; MCF-7_DOX2-9_ cells). At selection dose 7 (6.5 nM doxorubicin), MCF-7_DOX2-7_ cells did not exhibit statistically significant resistance to doxorubicin, despite the elevated expression of Bcl-2. It is possible that a low level of doxorubicin resistance (mediated by Bcl-2) was acquired at selection dose 7, but this could not be detected due to the limitations in the sensitivity of the clonogenic assay. However, Bcl-2 expression dramatically *decreased* during selection for resistance to higher doses of doxorubicin, co-incident with loss of ERα phosphorylation. While one would expect levels of an anti-apoptotic protein such as Bcl-2 to remain high or increase further at higher selection doses, the levels of this protein actually decreased. There was simply insufficient active ERα to drive its expression. The downregulation of Bcl-2 at higher selection doses would nevertheless activate another survival pathway (autophagy), which promotes the degradation of damaged organelles and greater survival from the generation of reactive oxygen species generated by doxorubicin [48]. Bcl-2 is a known inhibitor of autophagy through its ability to bind Beclin 1 (reviewed in [61]). Since Bcl-2 has been shown to be regulated by ERα [48], it stands to reason that ERα can act as an indirect regulator of the autophagic pathway. The downregulation of ERα activity would result in reduced Bcl-2 expression, resulting in disinhibition (activation) of the autophagic pathway. In addition to changes in Bcl-2 expression, cyclin D1 levels were observed to be reduced as active ERα levels fell. Since cyclin D1 promotes progression through the cell cycle, low cyclin D1 would be expected to slow cell cycle progression, permitting greater time for repair of doxorubicin-induced cellular damage.

The results from immunoblotting experiments suggest a clear correlation between the downregulation of ERα activity upon acquisition of doxorubicin resistance and changes in the expression of Bcl-2 and cyclin D1. However, this correlation need not necessarily mean the relationship is causative. We thus examined the effects of Artemisinin on Bcl-2 and cyclin D expression, since the drug is known to downregulate ERα expression, without inducing doxorubicin resistance [62]. As expected when ERα levels were reduced due to treatment with Artemisinin, Bcl-2 and cyclin D1 levels were also reduced as shown in previous studies. This suggests that the observed reduction in both cellular growth rate and saturation density as cells acquire anthracycline resistance may, in fact, be due to the loss of ERα protein levels and function, which in turn would reduce cell cycle progression and promote autophagic survival through negative effects on Cyclin D1 and Bcl-2 expression, respectively.

We further show in this study that while Akr1c3 and Akr1b10 might be expected to contribute to doxorubicin resistance through the hydroxylation/inactivation of the drug or through its ability to combat reactive oxygen species generated by doxorubcin, downregulation of Akr1c3 and/or Akr1b10 does not result in the restoration of doxorubicin sensitivity in MCF-7_DOX2-12_ cells. In addition, others have shown that doxorubicin does not appear to be a good substrate for the above aldoketoreductases in cells [49, 50]. However, we have also shown previously published studies that the aldo-keto reductase inhibitor β-cholanic acid can almost completely restore doxorubicin sensitivity to MCF-7_DOX2-12_ cells [13, 14]. This would suggest that β-cholanic acid may have a wider effect on doxorubicin cytotoxicity than simply inhibiting the aldo-keto reductases, a hypothesis now supported by observations that β-cholanic acid also promotes selective accumulation of doxorubicin into MCF-7_DOX2-12_ cells, but not MCF-7_CC12_ cells [Chewchuk et al., manuscript in preparation].

## Supporting information

S1 Fig Analysis of ER-β activity in doxorubicin-resistant cells. **(A)** Representative western blot of ER- β expression levels in dose selection 7–12 resistant and co-culture control MCF-7 cells. Primary antibody for ER-β was purchased from Santa Cruz Biotechnology (sc-8974). Blot is representative of 3 trials. **(B-D)** Q-PCR results for selected ER-β responsive genes in dose selections 7 and 12 with corresponding co-culture controls (n = 3), graphs represent fold expression relative to ribosomal protein S28 (RPS28) expression. Primers were purchased from Integrated DNA Technologies. **(B)** Relative expression of cyclin A1. Primer sequences for Cyclin A1were F: 5’- GCA CCC TGC TCG TCA CTT G -3’ R: 5’- CAG CCC CCA ATA AAA GAT CCA -3’, **(C)** Relative expression of HSD11B2. Primer sequences for HSD11B2 were F: 5’- CTG GCT GCT TCA AGA CAG AGT -3’ R: 5’- AGG CAG GTA GTA GTG GAT GAA -3’ and **(D)** Relative expression of TMOD1. Primer sequences for TMOD1 were F: 5’- CCG GTT CCA GCG TCA CA -3’ R: 5’- AGG AAA GGT CTG GGT TCC TAA GC -3’. No statistically significant changes were observed in overall protein levels of ER-β or in the expression of any of the tested ER-β responsive genes as a result of selection for resistance to Doxorubicin.
(TIF)

S2 Fig Analysis of ER-α knock-down in MCF-7 wild type cells by siRNA. siRNA was purchased from Life Technologies. Sequences correspond to catalogue numbers 4823 (ER-KD-3) and 4825 (ER-KD-5) **(A)** Q-PCR data for fold knock down of ER-α relative to scrambled control and was assayed in parallel with survival 24h post transfection. Graph is mean ± SEM of 5 trials. **(B)** Representative clonogenic survival curve for MCF-7 cells with ER-α knockdown or scrambled control showing no significant shift in IC50 associated with knockdown of ER-α relative to control. **(C)** Average IC50 values of MCF-7 cells with siRNA knockdown of ER-α as derived from replicate survival curves. Graph represents the mean ± SEM of 5 trials. No significant difference in IC50 values was observed for either ER-α knockdown condition relative to scrambled control.
(TIF)
