# Supplementary figures and images for: Alterations in estrogen signalling pathways upon acquisition of anthracycline resistance in breast tumor cells
Source: PLoS One. 2017 Feb 14;12(2):e0172244. doi: 10.1371/journal.pone.0172244 (PMC5308870; doi:10.1371/journal.pone.0172244)

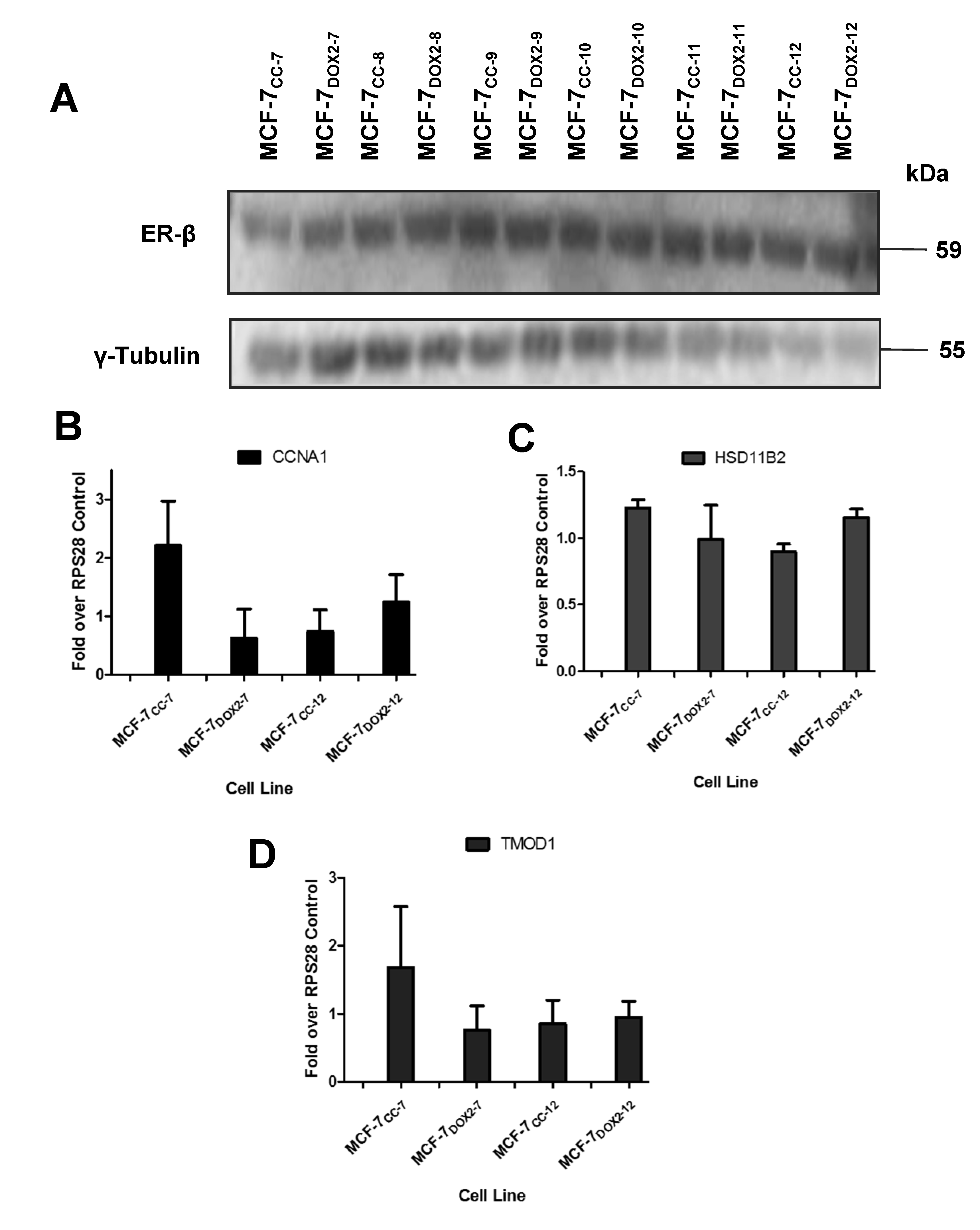

Supplement: S1 Fig — (A) Representative western blot of ER- β expression levels in dose selection 7–12 resistant and co-culture control MCF-7 cells. Primary antibody for ER-β was purchased from Santa Cruz Biotechnology (sc-8974). Blot is representative of 3 trials. (B-D) Q-PCR results for selected ER-β responsive genes in dose selections 7 and 12 with corresponding co-culture controls (n = 3), graphs represent fold expression relative to ribosomal protein S28 (RPS28) expression. Primers were purchased from Integrated DNA Technologies. (B) Relative expression of cyclin A1. Primer sequences for Cyclin A1were F: 5’- GCA CCC TGC TCG TCA CTT G -3’ R: 5’- CAG CCC CCA ATA AAA GAT CCA -3’, (C) Relative expression of HSD11B2. Primer sequences for HSD11B2 were F: 5’- CTG GCT GCT TCA AGA CAG AGT -3’ R: 5’- AGG CAG GTA GTA GTG GAT GAA -3’ and (D) Relative expression of TMOD1. Primer sequences for TMOD1 were F: 5’- CCG GTT CCA GCG TCA CA -3’ R: 5’- AGG AAA GGT CTG GGT TCC TAA GC -3’. No statistically significant changes were observed in overall protein levels of ER-β or in the expression of any of the tested ER-β responsive genes as a result of selection for resistance to Doxorubicin. (TIF) [file pone.0172244.s001.tif]

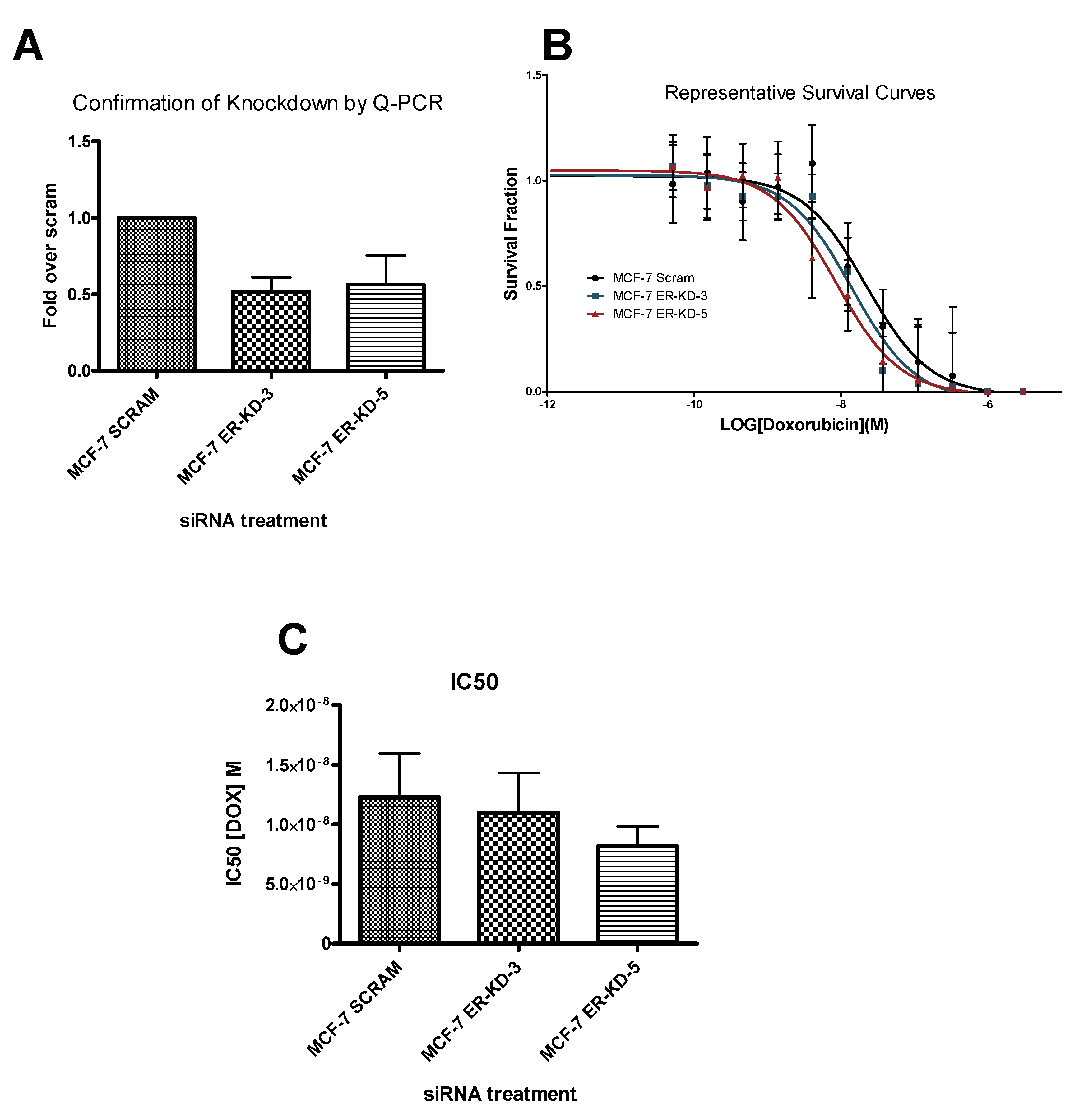

Supplement: S2 Fig — siRNA was purchased from Life Technologies. Sequences correspond to catalogue numbers 4823 (ER-KD-3) and 4825 (ER-KD-5) (A) Q-PCR data for fold knock down of ER-α relative to scrambled control and was assayed in parallel with survival 24h post transfection. Graph is mean ± SEM of 5 trials. (B) Representative clonogenic survival curve for MCF-7 cells with ER-α knockdown or scrambled control showing no significant shift in IC50 associated with knockdown of ER-α relative to control. (C) Average IC50 values of MCF-7 cells with siRNA knockdown of ER-α as derived from replicate survival curves. Graph represents the mean ± SEM of 5 trials. No significant difference in IC50 values was observed for either ER-α knockdown condition relative to scrambled control. (TIF) [file pone.0172244.s002.tif]
